# Supplementary material for: Switchable tribology of ferroelectrics
Source: Nat Commun. 2024 Jan 9;15:387. doi: 10.1038/s41467-023-44346-0 (PMC10776724; doi:10.1038/s41467-023-44346-0)
Supplement: Supplementary file 1 — Supplementary Information [file 41467_2023_44346_MOESM1_ESM.pdf]

Supplementary Information for

Switchable tribology of ferroelectrics

Seongwoo Cho[1,2*], Iaroslav Gaponenko[2,3], Kumara Cordero-Edwards[2], Jordi Barceló-Mercader[4], Irene Arias[4,5], Daeho Kim[1], Céline Lichtensteiger[2], Jiwon Yeom[1], Loïc Musy[2], Hyunji Kim[1], Seung Min Han[1], Gustau Catalan[6,7], Patrycja Paruch[2*] and Seungbum Hong[1,8*]

[1]Department of Materials Science and Engineering, Korea Advanced Institute of Science and Technology (KAIST); Daejeon 34141, Republic of Korea

[2]Department of Quantum Matter Physics, University of Geneva; 1211 Geneva, Switzerland

[3]G.W. Woodruff School of Mechanical Engineering, Georgia Institute of Technology; Atlanta, Georgia 30332, United States of America

[4]LaCàN - Mathematical and Computational Modeling, Polytechnic University of Catalonia; Barcelona 08034, Spain

[5]International Centre for Numerical Methods in Engineering (CIMNE); Barcelona 08034, Spain

[6]Catalan Institute of Nanoscience and Nanotechnology (ICN2), Campus Autonomous University of Barcelona; Bellaterra 08193, Spain

[7]Catalan Institution for Research and Advanced Studies (ICREA); Barcelona 08010, Catalonia

[8]KAIST Institute for NanoCentury (KINC), Korea Advanced Institute of Science and Technology (KAIST); Daejeon 34141, Republic of Korea

*Corresponding authors:

seongwoo.cho@unige.ch, patrycja.paruch@unige.ch, seungbum@kaist.ac.kr

Table of Contents

Supplementary text 4
1. Periodically poled lithium niobate (PPLN) 4
2. Effect of loading force on the asymmetric nano-tribology 4
3. Degradation of friction and wear 5
4. Effect of scan rate on the asymmetric nano-tribology 6
5. Computational model 7
6. Contact model 12
7. Flexoelectric contact simulations 13
8. *I*–*V* curve measurements 14
9. Asymmetric friction and wear in stoichiometric $LiNbO_3$ 14
10. Quantitative indentation depth comparison: nanoindentation of ferroelectric up and down crystals 15
11. Tomographic studies of $LiNbO_3$ nanopillars 16
12. Ferroelectric polarization-derived lithography of $LiNbO_3$ using wear asymmetry 16

Supplementary Figures 18
Fig. S1. Description of pristine single crystal periodically poled $LiNbO_3$ (PPLN) 18
Fig. S2. Self-cleaning of wear debris by AFM tip after milling scans 19
Fig. S3. Topography before and after mechanical grinding in Fig. 1 with the pristine PFM and friction images 20
Fig. S4. Surface roughness before and after milling scans in Fig. 1 21
Fig. S5. PFM amplitude before and after the mechanical grinding shown in Fig. 1 22
Fig. S6. Cross-validation of wear asymmetry using SEM 23
Fig. S7. Contact force optimization of asymmetric friction and wear 24
Fig. S8. Effect of increasing and decreasing loading force on asymmetric tribology using the same probe 25
Fig. S9. Friction and wear trend from the experiment in Fig. S8 26
Fig. S10. Simultaneous PFM imaging of PPLN with increasing loading force from 200 nN to 20 µN showing no transient domain switching during the application of high strain gradient 27
Fig. S11. Friction and wear degradation with continuous scans 28
Fig. S12. SEM images of the diamond probe before and after milling 29
Fig. S13. Effect of scan rate on asymmetric tribology 30
Fig. S14. Control experiments of scalable large-area milling of PPLN 32
Fig. S15. Scalable polarization-derived lithography of PZN-PT single crystals 33
Fig. S16. Geometrical parameters of the spherical indenter and axisymmetric problem statement with mechanical boundary conditions 34
Fig. S17. Validation of the contact model against the analytical model for linear elasticity 35

Fig. S18. Schematic of the axisymmetric three-dimensional model depicting the electric potential distribution upon indentation using a spherical AFM probe 36
Fig. S19. Polarization fields upon indentation induced by flexoelectricity and piezoelectricity for up and down ferroelectric domains, for $f = 10\ V$ 37
Fig. S20. The effect of flexoelectricity on indentation depth and contact radius in up and down domains 38
Fig. S21. Asymmetric indentation depth and contact area of ferroelectric up and down domains with different flexocoupling coefficients 39
Fig. S22. Electric potential distribution and indentation depth depending on electric ground condition 40
Fig. S23. *I-V* curve measurements on highly ordered pyrolytic graphite using six different probe types 41
Fig. S24. Tribological asymmetry with a metal-coated Si probe 42
Fig. S25. Temperature-dependent milling experiment on PPLN 43
Fig. S26. Asymmetric friction and wear of stoichiometric $LiNbO_3$ single crystal 44
Fig. S27. Nanoindentation of stoichiometric $LiNbO_3$ single crystals 45
Fig. S28. Switchable tribological asymmetry in PPLN 46
Fig. S29. Fabrication of nanopillars in Fig. 3 47
Fig. S30. Nanostructuring using switchable wear asymmetry of ferroelectric $LiNbO_3$ thin film 48
Fig. S31. Height, PFM phase and PFM amplitude images obtained after the electrical switching of up domains to down domains by applying 5 V to the AFM tip. 49
Fig. S32. Lattice-scale polarization-derived lithography. 50
Fig. S33. Demonstration of 3D nanostructure fabrication of a ferroelectric $LiNbO_3$ thin film 51
Fig. S34. Structural evolution during the 3D nanostructuring in Fig. S33 52
Fig. S35. Height and PFM phase with pristine background after 3D nanostructuring 53

Supplementary Tables 54
Table S1. Probe selection for the asymmetric wear of PPLN 54
Table S2. Ratio of friction in up and down domains for different values of the flexocoupling coefficient 55
Table S3. Indentation depth from loading curves in nanoindentation measurement using two different types of indenters 56

References 57

Supplementary Text

1. Periodically poled lithium niobate (PPLN)

Asymmetric friction and wear were observed in a z-cut PPLN single crystal. $LiNbO_3$ is a simple and ideal model system for probing asymmetric tribology because ferroelastic switching (e.g., switching from a c-domain to an a-domain or vice versa) is limited. Therefore, only up and down domains exist, even with a high strain gradient. Without the possible effects from a-domains in the crystal[1], we investigated the PPLN single crystal as schematically shown in Fig. S1a. Topography, PFM amplitude and phase were visualized by single frequency PFM imaging using a conductive diamond probe (NM-TC, Adama Innovations, Lot number: 009-013). Fig. S1b shows the flat surface of the pristine PPLN (roughness = 251.2 pm). PFM amplitude and phase (Figs. S1c and S1d) indicate uniaxial out-of-plane ferroelectric domains (up and down), where the domain width is approximately 5 μm with periodic stripes.

2. Effect of loading force on the asymmetric nano-tribology

With a pristine single-crystalline conductive diamond tip (NM-TC), we optimized the asymmetric wear in four different regions with the same polarity. We scanned each area 10 times with loading forces from 2.5 to 20 μN, as shown in Fig. S7a. Fig. S7a shows the height after ten scans performed with different loading forces, and Fig. S7b shows the PFM phase image, confirming the stability of the domain configuration after milling scans. We found an optimal loading force regime for asymmetric wear. If the loading force is low (i.e., below 2.5 μN), no notable wear of the crystal is observed (the wear depth calculated by subtracting the average height value of the milled surface from the pristine background surface is -16.0 pm, which is much lower than sample roughness). If the loading force is in the optimum regime (from 5 to 10 μN), asymmetric wear occurs, and the up and down domains have different heights, with higher average friction in up domains ($Friction^{up}/Friction^{down}$ is 1.057 at 5 μN and 1.051 at 10 μN). In the 10 μN case, the left side of the worn area experiences partial breaking, indicating a higher loading force applied in this over-scanned region. If the loading force is high (i.e., approximately 20 μN), the resulting topography is dominated by milling with material fracture rather than asymmetric wear. Therefore, the loading force should be higher than the force required to initiate the wear but lower than the material fracture regime for asymmetric milling.

Figs. S8 and S9 also depict the effects of increasing and decreasing loading force on friction asymmetry and wear behavior. With a pristine diamond probe, each region was milled four times with a loading force starting from 1 to 10 µN in 10 different regions with the same polarity (Fig. S8a). We observe no significant wear in the regions milled from 1 to 3 µN, but do observe asymmetric wear in regions milled from 4 to 10 µN. Fig. S8b shows the height image obtained in an analogous manner but this time decreasing the loading force from 10 to 1 µN using the same tip, and we observe asymmetric wear between 10 and 7 µN. Fig. S8c shows the PFM phase in the same region as in Fig. S8b before millings, which indicates alternating up and down domains. We believe that the origin of this hysteretic behavior is probe degradation, which is further described in Figs. S11 and S12.

The height after milling of the PPLN surface is also seen in the line profiles in Figs. S9a and S9b. In heavily milled regions (4–10 µN in Fig. S8a and 7–10 µN in Fig. S8b), the wear depth of the down domains (purple) is always lower than that of the up domains (orange). Figs. S9c and S9d indicate friction signals versus scan numbers with height during specific scans. As can be seen in the height images in Fig. S9c, the asymmetric wear starts during the 2nd scan at 4 µN. We observe the friction signal jumps up from 2nd scan at 4 µN because of higher resistance to the AFM tip motion during wear. In Fig. S9d, the asymmetric wear starts from the 3rd scan at 7 µN, and the friction signal also rapidly increases at the 3rd scan at 7 µN, which implies a strong correlation between friction and asymmetric wear behavior.

Furthermore, no transient flexoelectric switching occurred during the high force application as can be seen in Fig. S10. We simultaneously visualized ferroelectric domains with increasing loading forces from 200 nN to 20 µN, but we observed no flexoelectric switching from up to down domain even with loading forces (e.g., 15 and 20 µN) in the material fracture regime.

3. Degradation of friction and wear

The asymmetric friction and wear rate show degradation with continuous milling scans, as can be seen in Fig. S11. Fig. S11a shows the PFM phase image before milling scans. Fig. S11b shows the resulting height after 20 milling scans in each region from region 1 (R1) to region 4 (R4), with the same milling conditions (loading force of 5 µN, scan rate of 1.95 Hz, controlled environmental condition of 20°C and relative humidity of 20%). Even under the same conditions, the average height difference in a pristine area shows a 61.1% decrease from

R1 (96.5 nm) to R4 (37.5 nm). Figs. S11c and S11d show the wear depth in each region and especially the height difference between up (orange) and down domains (purple) in Fig. S11d. Fig. S11e shows the degrading behavior of friction signals, where the first friction signal in each region showing relatively lower friction might originate from the skin layer or adsorbates on the surface during the first milling scan. The average asymmetric friction tends to deteriorate with scanning as demonstrated by the signal in each region, which reveals a 35.9% decrease from R1 to R4. The correlation between the average friction and wear in each region shows a linear degradation from R1 to R4, as shown in Fig. S11f, suggesting that the mechanism possibly originated from asymmetric mechanical stiffness differences in up and down domains, because the quality of mechanical contact determines the wear efficiency. Furthermore, this correlation implies that the optimal loading force for surface milling changes with continuous milling. Scanning electron microscope (SEM) images of the pristine tip and the same tip after milling (Fig. S12) exhibit clear differences, with wear debris attached to the tip side after the milling scans making non-ideal indentation between the tip and the sample.

4. Effect of scan rate on the asymmetric nano-tribology

The effect of the scan rate on nano-tribology was investigated in the PPLN sample using a pristine single crystalline conductive diamond tip (NM-TC, Adama Innovations, Lot number: 009-013). Four different regions with the same polarity in each image were milled 10 times. First, the scan rate is increased from 1.26 to 9.77 Hz in four regions with a loading force of 5 μN, as can be seen in Fig. S13a. The wear depth decreases with an increasing scan rate. Using the same probe immediately after the experiment in Fig. S13a, with a decreasing scan rate from 9.77 to 1.26 Hz at 5 μN, we observe drastically reduced wear depth (Fig. S13b). We then increased the loading force from 5 to 8 μN with a decreasing order of scan rate from 9.77 to 1.26 Hz, and observed degrading behavior, concluding that the tribological asymmetry is dominated by a change in the loading force and continuous degradation during the milling, rather than a change in scan rate (Fig. S13c). PFM phase images after three sets of experiments show stable domain configuration after experiments shown in Figs. S13d–f.

Fig. S13g plots friction vs. scan rate. Even with high standard deviations in the friction signals of both the up and down domains, the average friction signal is always higher in the up domain than the down domain. Figs. S13h and S13i show the wear depth vs. scan rate in Figs.

S13a and S13c, respectively. The wear depth again decreases with scan order, not with the scan rate change.

5. Computational model

We follow the linear continuum model of piezoelectricity augmented with flexoelectricity[2]. The electromechanical enthalpy density of a dielectric solid exhibiting piezoelectricity (without polarization reversal) and flexoelectricity considered here, in terms of the strain $\boldsymbol{\varepsilon}$, the strain gradient $\nabla\boldsymbol{\varepsilon}$, and the electric field $\boldsymbol{E}$, is given by

$$\Psi(\boldsymbol{\varepsilon},\nabla\boldsymbol{\varepsilon},\boldsymbol{E}) = \frac{1}{2}C_{ijkl}\varepsilon_{ij}\varepsilon_{kl} - e_{ijk}E_i\varepsilon_{kl} - \mu_{ijkl}E_i\varepsilon_{jk,l} - \frac{1}{2}\kappa_{ij}E_iE_j + \frac{1}{2}h_{ijklmn}\varepsilon_{ij,k}\varepsilon_{lm,n} \quad \text{(S1)}$$

where $\boldsymbol{C}$ is the fourth-rank elasticity tensor, $\boldsymbol{e}$ is the third-rank piezoelectricity tensor, $\boldsymbol{\mu}$ is the fourth-rank flexoelectricity tensor, $\boldsymbol{\kappa}$ is the second-rank dielectricity tensor and $\boldsymbol{h}$ is the sixth-rank strain gradient elasticity tensor. The strain gradient elasticity term is required to guarantee the thermodynamic stability of the model in the presence of flexoelectricity[3-5]. In this formulation, the remanent state of the piezoelectric material is taken as the reference state, relative to which strain $\boldsymbol{\varepsilon}$ and polarization $\boldsymbol{p}$ are defined. Thus, the poling (up or down) of the piezoelectric material is encoded in the polarity of the piezoelectric tensor $\boldsymbol{e}$[6]. The associated free enthalpy of the system is given by

$$\Pi[\boldsymbol{u},\phi] = \int_\Omega \Psi \, \mathrm{d}\Omega - W^{ext}, \qquad \text{(S2)}$$

where $\boldsymbol{u}$ is the displacement, ϕ is the electric potential with $\boldsymbol{E} = -\nabla\phi$ the electric field, Ω denotes the solid domain, and W^{ext} is the work of external forces and charges. The mechanical equilibrium and electrostatic governing equations follow from the variational principle $(\boldsymbol{u}^*,\phi^*) = \arg \min_{\boldsymbol{u}} \max_{\phi} \Pi[\boldsymbol{u},\phi]$ as Ref.[7]

$$\begin{cases} (\hat{\sigma}_{ij} - \tilde{\sigma}_{ijk,k})_{,j} + b_i = 0, & \text{in } \Omega \\ D_{l,l} - q = 0, & \text{in } \Omega, \end{cases} \qquad \text{(S3)}$$

where $\boldsymbol{b}$ is the external body forces per unit volume, q the external free charges per unit volume, and the stress $\hat{\boldsymbol{\sigma}}$, double stress $\tilde{\boldsymbol{\sigma}}$, and electric displacement $\boldsymbol{D}$ are

$$\hat{\sigma}_{ij}(\boldsymbol{u},\phi)=\left.\frac{\partial\Psi[\boldsymbol{\varepsilon},\boldsymbol{\nabla\varepsilon},\boldsymbol{E}]}{\partial\varepsilon_{ij}}\right|_{\substack{\boldsymbol{\nabla\varepsilon}\\ \boldsymbol{E}}}=C_{ijkl}E_{kl}-e_{ijk}E_i, \tag{S4}$$

$$\tilde{\sigma}_{ijk}(\boldsymbol{u},\phi)=\left.\frac{\partial\Psi[\boldsymbol{\varepsilon},\boldsymbol{\nabla\varepsilon},\boldsymbol{E}]}{\partial\varepsilon_{ij,k}}\right|_{\substack{\boldsymbol{\varepsilon}\\ \boldsymbol{E}}}=h_{ijklmn}E_{lm,n}-\mu_{ijkl}E_i, \tag{S5}$$

$$D_l(\boldsymbol{u},\phi)=-\left.\frac{\partial\Psi[\boldsymbol{\varepsilon},\boldsymbol{\nabla\varepsilon},\boldsymbol{E}]}{\partial E_l}\right|_{\substack{\boldsymbol{\nabla\varepsilon}\\ \boldsymbol{E}}}=\kappa_{lm}E_m+e_{lij}E_{ij}+\mu_{lijk}E_{ij,k}. \tag{S6}$$

Note that the physical stress is $\boldsymbol{\sigma}=\hat{\boldsymbol{\sigma}}-\boldsymbol{\nabla}\tilde{\boldsymbol{\sigma}}$. Equations S3 are subject to appropriate boundary conditions, which for this coupled higher-order problem are slightly involved. We refer the reader to Ref. [2] for a complete account and only present here those used in our present calculations. Boundary conditions can be split into Dirichlet conditions, prescribing the unknown fields in portions of the boundary $\partial\Omega$, and Neumann conditions, prescribing the corresponding work-conjugate quantities. Dirichlet and Neumann conditions for each field are mutually exclusive at a given location of the boundary. Dirichlet conditions prescribe the displacement on Γ_u and the electric potential on Γ_ϕ, both subsets of $\partial\Omega$, as

$$u_i=\bar{u}_i \ \text{ on } \ \Gamma_u \tag{S7a}$$

$$\phi=\bar{\phi} \ \text{ on } \ \Gamma_\phi \tag{S7b}$$

where the overline indicates a known prescribed quantity. To specify the Neumann boundary conditions, we obtain the mechanical traction $\boldsymbol{t}(\boldsymbol{u},\phi)$ and the electric charge density $w(\boldsymbol{u},\phi)$ on the boundary of the domain as

$$t_i(\boldsymbol{u},\phi) = \left(\hat{\sigma}_{ij} - \tilde{\sigma}_{ijk,k} - \tilde{\sigma}_{ikj,l}(\delta_{lk} - n_l n_k)\right) n_j + \tilde{\sigma}_{ijk}\widetilde{N}_{jk} \quad \text{on } \partial\Omega, \qquad (S8a)$$

$$w(\boldsymbol{u},\phi) = -\widehat{D}_l n_l \qquad (S8b)$$

where $\widetilde{N}_{ij} = -n_{i,l}(\delta_{lj} - n_l n_j) + n_{f,g}(\delta_{fg} - n_f n_g) n_i n_j$ and $\mathbf{n}$ is the unit outer normal vector[7,8]. The following describes mechanical and electrical Neumann boundary conditions

$$t_i = \bar{t}_i \text{ on } \Gamma_t, \qquad (S9a)$$

$$w = \bar{w} \text{ on } \Gamma_w \qquad (S9b)$$

Because the enthalpy depends on the second derivatives of the displacement, one also needs to consider higher-order and corner conditions[7]. Here, we adopt the most natural and straightforward choice of imposing homogeneous Neumann higher-order and corner conditions[8].

Focusing on the indentation simulations, we consider a cylindrical domain, as shown in Fig. S16, with the indenter at the center of the top surface. Assuming axisymmetry, we restrict our analysis to a rectangular domain in the $r - z$ plane as shown in Fig. S16. The unknowns are the two components of the displacement field, u_r and u_z and the electric potential ϕ. We refer the reader to Refs. [9,10] for a detailed account of the strains and strain gradients in an axisymmetric formulation. We numerically solve the governing equations using a B-Spline finite element method as described in Refs. [2,7].

In our simulations, we set external body forces and distributed charges to zero, $\boldsymbol{b} = 0$ and $q = 0$. At the bottom part of the boundary, given by $z = 0$, we fix the electric potential and the vertical component of the displacement to zero, $u_z = 0$ and $\phi = 0$. At the left part of the boundary, given by $r = 0$, we fix the radial component of the displacement to zero, $u_r = 0$. At the top-left corner of the domain, we impose the contact conditions described below. All other boundary conditions are traction-free ($t_z = 0$ everywhere except at $z = 0$, $t_r = 0$ everywhere except at $r = 0$) and zero surface charge density ($w = 0$ everywhere except at $z = 0$). Because of axisymmetry and the higher-order nature of the problem, we also impose

that $\partial_r u_r = 0$ and $\partial_r u_z = 0$ at $r = 0$. To model the up or down poling of the material, we changed the polarity of the piezoelectric tensor accordingly[11].

The electrical boundary conditions correspond to an open circuit. To test the robustness of the asymmetrical response depending on polarization direction in the crystal, we alternatively considered closed circuit conditions by either setting $\phi = 0$ on the top boundary $(z = H)$ or only on the part of this boundary in contact with the indenter. This result is shown in Fig. S22.

Regarding material parameters, all values have been chosen to fit the behavior of PPLN, while reducing the number of uncertain material parameters and simulation costs by considering isotropic material response and axisymmetric conditions. The material parameters for elasticity, dielectricity and piezoelectricity are taken from an open database of computed material properties[12]. We also used a wide range of flexocoupling tensor values in the simulation including theoretical and experimental values. The flexoelectric coupling coefficients of 1 V and 10 V are based on Kogan's estimate of the flexocoupling tensor[13,14]. The flexoelectric coupling coefficients of 40 V and 54 V are used based on the experimental values from Ref. [15].

We use an isotropic elasticity tensor defined in terms of the Young modulus E and Poisson ratio ν as

$$\begin{aligned} &C_{iiii} = C_L, && i = 1,2,3; \\ &C_{iijj} = C_T, && i,j = 1,2,3 | i \neq j; \\ &C_{ijij} = C_{ijji} = C_S, && i,j = 1,2,3 | i \neq j; \end{aligned} \qquad \text{(S10)}$$

where the parameters C_L, C_S and C_T are

$$C_L := \frac{E(1-\nu)}{(1+\nu)(1-2\nu)}, \quad C_T := \frac{E\nu}{(1+\nu)(1-2\nu)}, \quad C_S := \frac{E}{2(1+\nu)}, \qquad \text{(S11)}$$

with $E = 146$ GPa and $\nu = 0.26$.

We choose an isotropic simplified form for the strain gradient tensor[2,7] depending on the Young's modulus E, the Poisson ratio ν and the internal length scale ℓ as

$$h_{iikiik} = \ell^2 C_L, \qquad i,k = 1,2,3;$$
$$h_{iikjjk} = \ell^2 C_T, \qquad i,j,k = 1,2,3|i \neq j;$$
$$h_{ijkijk} = h_{ijkjik} = \ell^2 C_S, \qquad i,j,k = 1,2,3|i \neq j; \qquad \text{(S12)}$$

with $\ell = 10$ nm.

We use an isotropic dielectricity tensor, κ, which depends on a parameter as

$$\kappa_{ii} = 45.07 \ nC/Vm, \qquad \kappa_{ij} = 0 \qquad i,j = 1,2,3. \quad \text{(S13)}$$

Piezoelectricity is represented by the polar third-order tensor $\boldsymbol{e}$. For a material with principal direction x_1, the piezoelectric tensor $\boldsymbol{e}^{<x_1>}$ is

$$e_{111}^{<x_1>} = 2.538 \ C/m^2 \ ;$$
$$e_{1jj}^{<x_1>} = 2.932 \ C/m^2 \ ; \qquad j = 2,3;$$
$$e_{j1j}^{<x_1>} = e_{jj1}^{<x_1>} = 0.0874 \ C/m^2 \ ; \qquad j = 2.3. \qquad \text{(S14)}$$

The piezoelectric tensor oriented in an arbitrary direction is obtained by rotating $\boldsymbol{e}^{<x_1>}$.

Isotropic cubic flexoelectricity has been chosen, with the flexoelectric tensor, μ, depending on two independent components, μ_L and μ_T. Here, we let $\mu_L = \mu_T = \kappa \ f$, where f is the flexocoupling coefficient. In simulations, four different values of the flexocoupling coefficient have been considered, f = 1, 10, 40 and 54 V. The components of the flexoelectric tensor $\boldsymbol{\mu}^{<x>}$ of a material oriented in the Cartesian axes are the following:

$$\mu_{iiii}^{<x>} = \kappa \ f, \qquad i = 1,2,3;$$
$$\mu_{ijji}^{<x>} = \kappa \ f, \qquad i,j = 1,2,3|i \neq j;$$
$$\mu_{iijj}^{<x>} = \mu_{ijij}^{<x>} = 0, \qquad i,j = 1,2,3|i \neq j. \qquad \text{(S15)}$$

6. Contact model

The Signorini-Hertz-Moreau model[16,17] for a pair of points in contact $(x; x_0)$ gives

$$g_n \leq 0, \qquad \sigma_n = \boldsymbol{n} \cdot \boldsymbol{\sigma} \cdot \boldsymbol{n}, \qquad g_n \sigma_n = 0 \quad on \quad \Gamma_c \qquad (S16)$$

where **n** is the normal vector to the contact boundary Γ_c and g_n is the gap function described as

$$g_n = \boldsymbol{u} \cdot \boldsymbol{n} + (\boldsymbol{x} - \boldsymbol{x}_0) \cdot \boldsymbol{n} \qquad (S17)$$

with x being a point on the flat surface of contact and x_0 the corresponding point of the rigid cone that is coming into contact. To enforce the non-penetration constraint, we add an extra surface energy term penalizing deviation from the conditions in Equations S16, yielding the final total enthalpy as

$$\overline{\Pi} = \Pi + \int_{\Gamma_c} \frac{1}{2} \beta \langle g_n \rangle^2 d\Gamma\,; \qquad (S18)$$

where β is a penalty parameter and $\langle A \rangle$ is the Macaulay bracket which returns the argument itself if the argument is positive and 0 otherwise. The rotational symmetry of the spherical tip and sample allows the use of the two-dimensional axisymmetric model. The contact force F is then obtained as

$$F = 2\pi \int_0^{R_c} \beta \langle g_n \rangle r dr. \quad (S19)$$

We verify the model against the classical spherical model in Ref. [18], for the case of an elastic solid with vanishing piezoelectricity $\boldsymbol{e}$, flexoelectricity $\boldsymbol{\mu}$, dielectricity $\boldsymbol{\kappa}$ and strain gradient elasticity $\boldsymbol{h}$ (Equation S1). We perform simulations for different vertical positions of the tip and compute the magnitude of the contact force and the contact radius. The relation between the indentation depth d and the contact radius R_c is given by

$$d = R_c \tanh^{-1}\left(\frac{R_c}{R}\right), \quad (S20)$$

where $R = 10$ nm corresponds to the nominal value provided by the manufacturer. The geometrical parameters of the simulation are shown in Fig. S16. The relation between the applied force F and the contact radius R_c is given by

$$F = E^* R^2 \left(\left(1 + \frac{R_c^2}{R^2} \right) \tanh^{-1} \left(\frac{R_c}{R} \right) - \frac{R_c}{R} \right), \qquad (S21)$$

where $E^* = \frac{E}{1-\nu^2}$, with $E = 146$ GPa and $\nu = 0.26$ in this simulation. We have considered a rectangular elastic solid of length $L = 40$ nm and height $H = 20$ nm in Fig. S16. We find perfect agreement between the theoretical model and the computational one, as shown in Fig. S17.

7. Flexoelectric contact simulations

Flexoelectricity breaks the mechanical symmetry and generates a noticeable difference in the response for up and down domains. This is further illustrated in Fig. S19, where the flexoelectrically induced polarization and piezoelectric polarization are plotted separately, for the case of upward and downward-polarized domains. While flexoelectric polarization is largely independent of the direction of ferroelectric polarization, piezoelectric polarization has opposite signs for up and down domains. Hence, their combination is the origin of the observed asymmetric response. Fig. S20 shows plots of the indentation depth and contact radius as functions of applied force. Apparently, in the absence of flexoelectricity, both quantities coincide for upward and downward-polarized domains, as expected. As indicated by previous studies on electromechanical response and fracture in dielectrics[14,15], the asymmetry resulting from the interaction of flexoelectricity and piezoelectricity is observed only in a range where these two mechanisms compete in magnitude. If either of them clearly dominates the other, i.e., for very small or very large flexoelectricity with respect to piezoelectricity, the asymmetry disappears, as the response of each mechanism is not asymmetric by itself. Within the range where flexoelectric and piezoelectric polarizations are comparable in magnitude, the observed difference in the response for up and down domains depends on the magnitude of flexoelectric parameters, being larger for stronger flexoelectricity (Fig. S21).

8. *I–V* curve measurements

To select a probe with negligible conductivity for the mechanism study of asymmetric tribology, we performed *I–V* curve measurements on a highly ordered pyrolytic graphite sample using six different types of probes: a Pt/Ir-coated Si probe (EFM, NanoWorld), diamond-coated Si probes (DT-NCHR and CDT-NCHR, Nanosensors), a diamond-like-carbon (DLC)-coated probe (HQ:NSC16/HARD/Al BS, MikroMasch) and single crystalline diamond probes (NM-TC, Adama Innovations and D300, SCD Probes). EFM, DT-NCHR, CDT-NCHR and NM-TC show conductive behavior as can be seen in Figs. S23a and S23b. DLC-coated probe and D300 probe show almost negligible current, but a closer look at *I–V* curves in Fig. S23c shows that D300 has the most negligible conductivity. D300 shows this insulating characteristic because the single crystalline diamond is attached to the cantilever with non-conductive glue. The probe selection for the asymmetric milling of PPLN is summarized with more details in Table S1. We successfully demonstrated asymmetric milling with five diamond probes, the DLC-coated probe and the Pt/Ti-coated Si Probe with high a spring constant (Spark 350-Pt, NuNano), but not with the Pt/Ir-coated Si probe with a low spring constant.

9. Asymmetric friction and wear in stoichiometric $LiNbO_3$

A third possible mechanism at the origin of the asymmetric tribology response is that the asymmetry is defect mediated. Asymmetric friction can originate from a defect concentration difference between up and down domains as in p-n junctions, which show varying friction because of charge depletion and accumulation[19]. The congruent PPLN used in this study is known to have relatively high densities of defects[20]. To evaluate their possible contribution, we investigated the wear effects in stoichiometric $LiNbO_3$, where the defect density is lower than in the congruent composition. Figs. S26a–c show height, PFM phase and amplitude before milling with naturally formed up and down domains in the sample scan region. Height and friction differences are observed between up and down domains during the milling scans shown in Figs. S26d (topography) and S26e (friction). During the milling scans, the friction is higher in up domains than in down domains. As a result of 10 milling scans at a loading force of 5 μN and a scan rate of 4.88 Hz, we also observe higher topography in the down domain (Fig. S26f), showing no significant difference between PPLN and stoichiometric $LiNbO_3$. Stable polarization configuration after milling scans is shown in the PFM phase and

amplitude images in Figs. S26g and S26h. Therefore, we conclude that the presence of pre-existing defects is not responsible for the observed asymmetry.

10. Quantitative indentation depth comparison: nanoindentation of ferroelectric up and down crystals

Previous study showed that ferroelectric up domains have higher hardness than ferroelectric down domains[21]. Wear rate and hardness show negative relation in most materials system[22]. This means that harder domains are less mechanically milled. However, from our experiments and simulations, ferroelectric up domains (harder domains) show higher mechanical wear rate. To elucidate this anomalous tribology of ferroelectrics, we conducted quantitative comparison of indentation depth via nanoindentation measurements on stoichiometric $LiNbO_3$ crystals depending on the out-of-plane polarization configuration.

Nanoindentation measurements were carried out using the iMicro (Nanomechanics, Inc.) nanoindentation system fitted with both spherical and Berkovich indenters. The strain fields with two different indenters (spherical and Berkovich) are expected to be different because of the different tip geometry; however, we assume that the Berkovich indenter generates a stronger strain field than the spherical indenter. Indentation was performed using continuous stiffness measurement (CSM) mode under load control test with a maximum load of 3 mN and 10 mN using the spherical and Berkovich indenters, respectively. Crack propagations were observed at the sharp corners of the Berkovich tip indentation, whereas elastic deformation was observed for spherical tip indentation. Fig. S27a shows higher hardness (1.82 ± 0.55 %) in ferroelectric up crystals using the Berkovich tip which exhibits the same trend in a previous fracture study of ferroelectrics[15]. Fig. S27b shows loading and unloading indentation curve of up and down crystals using the Berkovich indenter where ferroelectric down domains show higher indentation depth than ferroelectric up domains, which is the opposite to our experimental and simulation results. However, loading and unloading indentation curves of the spherical indenter (Fig. S27c) show higher indentation depth in ferroelectric up domains. This opposite trend may originate from the more pronounced crack propagation in ferroelectric down domains, which can be assumed from the previous study[15]. Averaged indentation depth is shown in Table S3 for each case, where the indentation depth is higher in ferroelectric up domains when tested with the spherical indenter tip, but the depth is higher for the down domains when tested with the Berkovich tip. The two opposite results in Fig. S27 and Table S3 indicate that the asymmetric tribology, where ferroelectric up domain

shows higher tribological responses than down domains, is only observable in a flexoelectrically optimized regime by using a spherical nanoindenter or AFM tip. In summary, ferroelectrics have asymmetric mechanical and tribological properties depending on the polarization orientation. Furthermore, even in the same polarization direction, nanomechanical and tribological behavior is controllable by adjusting the amount of strain gradient or stress applied to the crystals.

11. Tomographic studies of $LiNbO_3$ nanopillars

We note that up domains are visible in the core of the pillars after prolonged structuring of around 60 milling scans (see Supplementary Fig. 29e). This feature observed in $LiNbO_3$ nanodomain structures is a product of the electrostatic boundary conditions at the tip of the growing domain during initial switching, resulting in incomplete polarization reversal through the single crystal[23-25]. This core domain can place a limit on the pillar height, which can be achieved by nanodomain switching. As seen in Figs. 3d and 3e, while the pillars are still fully capped by a down domain (first 30 millings), the pillar height increases with milling in a linear fashion, similar to what we observed for the wide periodic domains fully penetrating the PPLN single crystal. As this cap is gradually worn through, and the up-polarized core is revealed, the wear rate of the pillar increases and becomes comparable to that of the up-oriented background state (Supplementary Fig. 29e). However, we also note that probe degradation, contact geometry, and complex interactions between the probe and the sample during milling and PFM scans can also influence the wear rate and nanostructuring.

12. Ferroelectric polarization-derived lithography using wear asymmetry

We found that our polarization-determined structuring could be demonstrated at reduced dimensions such as in 100 nm of $LiNbO_3$ thin film (NanoLN). To allow easily controllable domain patterning, we used a thinner $LiNbO_3$ fabricated by the ion slicing method. The pristine domain configuration is upward. Fig. S30 shows that facile fabrication without any chemicals or photomask is realized after the artificial decoration of the thin film with the text “FERRO” with down domains, and “LITHO” with up domains before milling. We used a Pt-coated Si probe (HQ:DPER-XSC11, MikroMasch) to switch the domain, and then changed to a single crystalline diamond probe (NM-TC, Adama Innovations, Lot number: 009-013) to prevent tip contamination. Multiple mechanical milling scans using a diamond probe create a

height difference between the up and down domains. As shown in Fig. S30d, the friction in the up domain is still higher than the down domain during the milling scan at a loading force of 12.5 μN, with a friction ratio close to the simulation values in Fig. 2g. Figs. 3f–i show the nanostructures presented in Fig. S30 with a pristine background region, which clearly indicate the height difference between patterned up and down domains and the pristine surface. Fig. S33 shows the 3D fabrication procedure for ferroelectric $LiNbO_3$ by repeated switching and milling. The loading force is gradually increased from 5 to 25 μN to maintain wear efficiency at a scan speed of 4.88 Hz. Fig. S34 shows the evolution of the topography during the 3D nanostructuring in Fig. S33.

Supplementary Figures

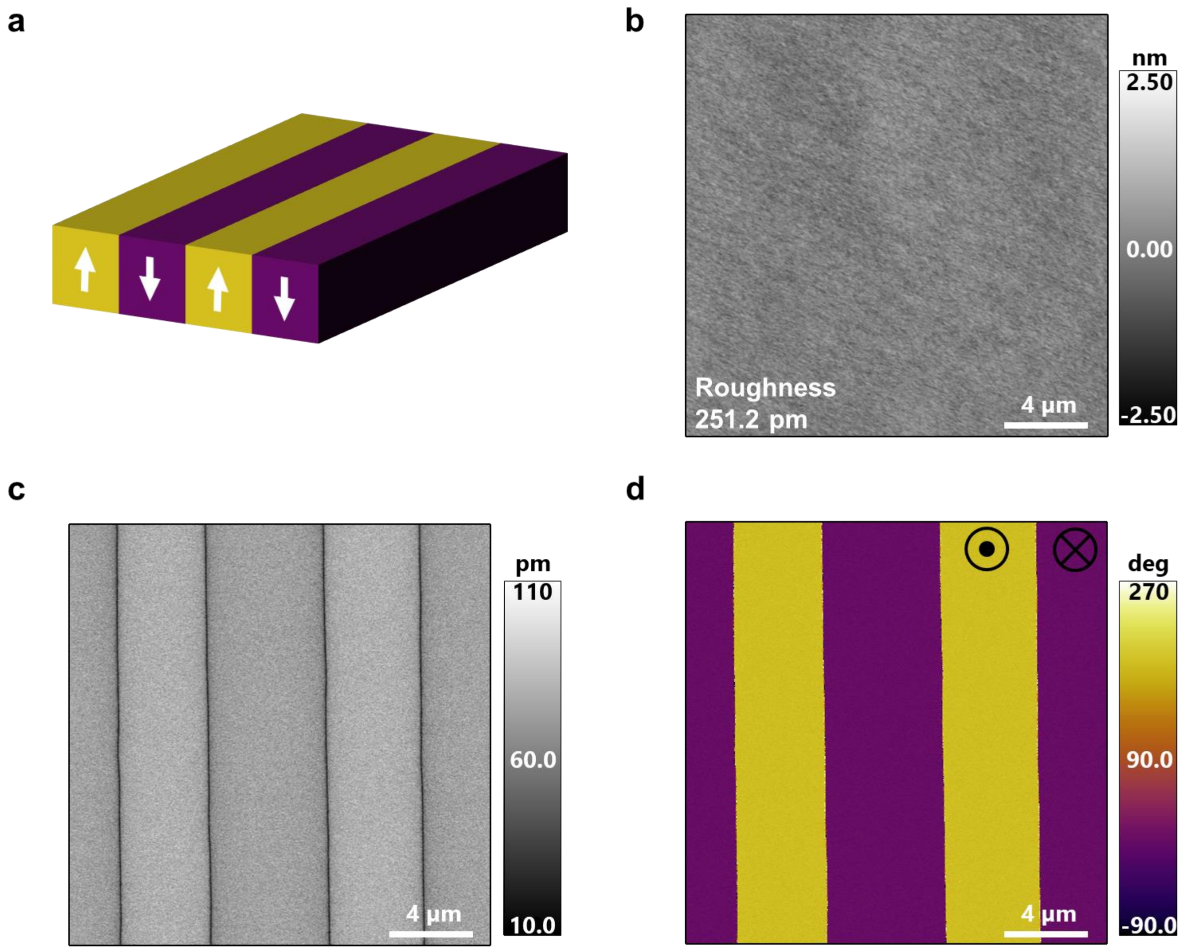

Fig. S1. Description of pristine single crystal periodically poled $LiNbO_3$ (PPLN). **(a)** Schematic of pristine z-cut PPLN, which has uniaxial out-of-plane ferroelectric domains (up and down polarity), **(b)** height, **(c)** PFM amplitude and **(d)** PFM phase of pristine PPLN. The height shows a flat morphology of the polished surface of the crystal, and PFM amplitude and phase show the stable alternative orientation of polarization, visualized by off-resonance single frequency PFM at 17 kHz. The domain width is around 5 μm with periodic stripes.

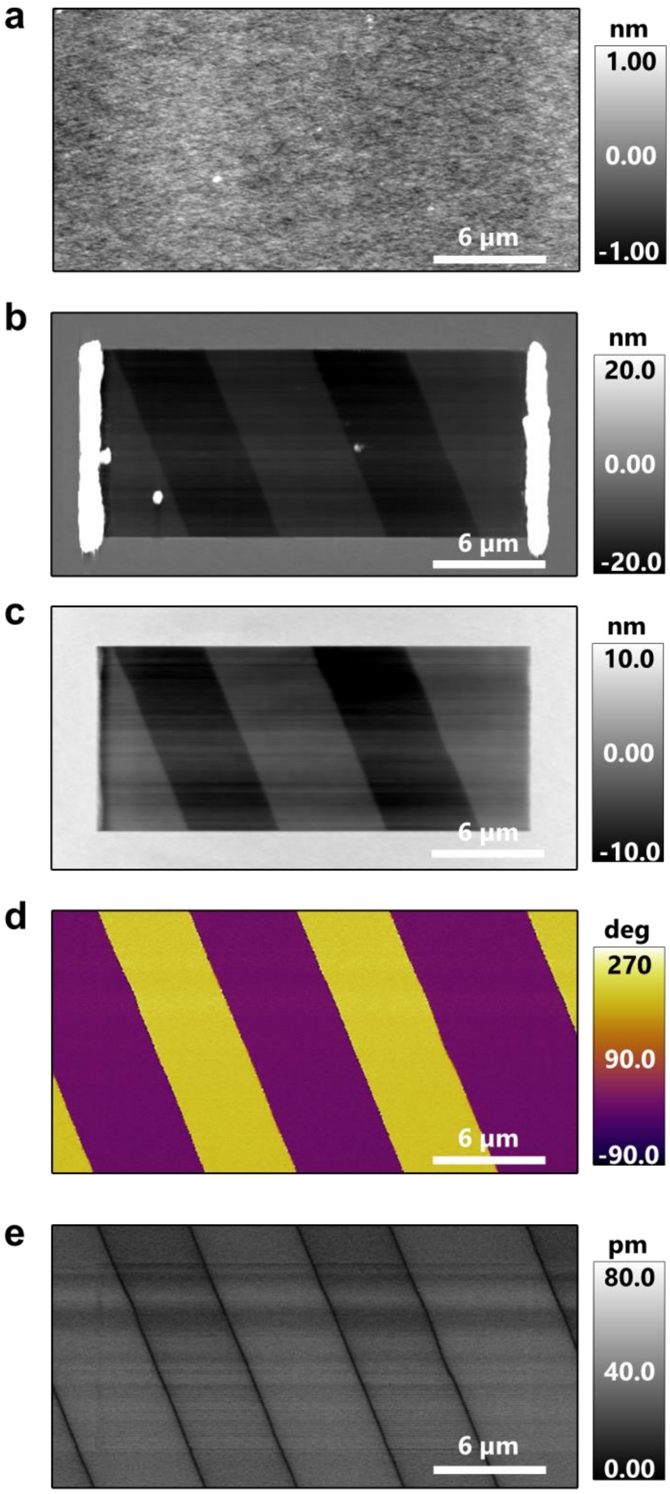

Fig. S2. Self-cleaning of wear debris by AFM tip after milling scans. (a) Height of pristine PPLN surface, **(b)** Height after continuous 40 milling scans at 8 μN. **(c)** Height after surface cleaning by AFM tip with 10 continuous contact scans at much lower loading force than that of milling. **(d)** PFM phase and **(e)** PFM amplitude after cleaning.

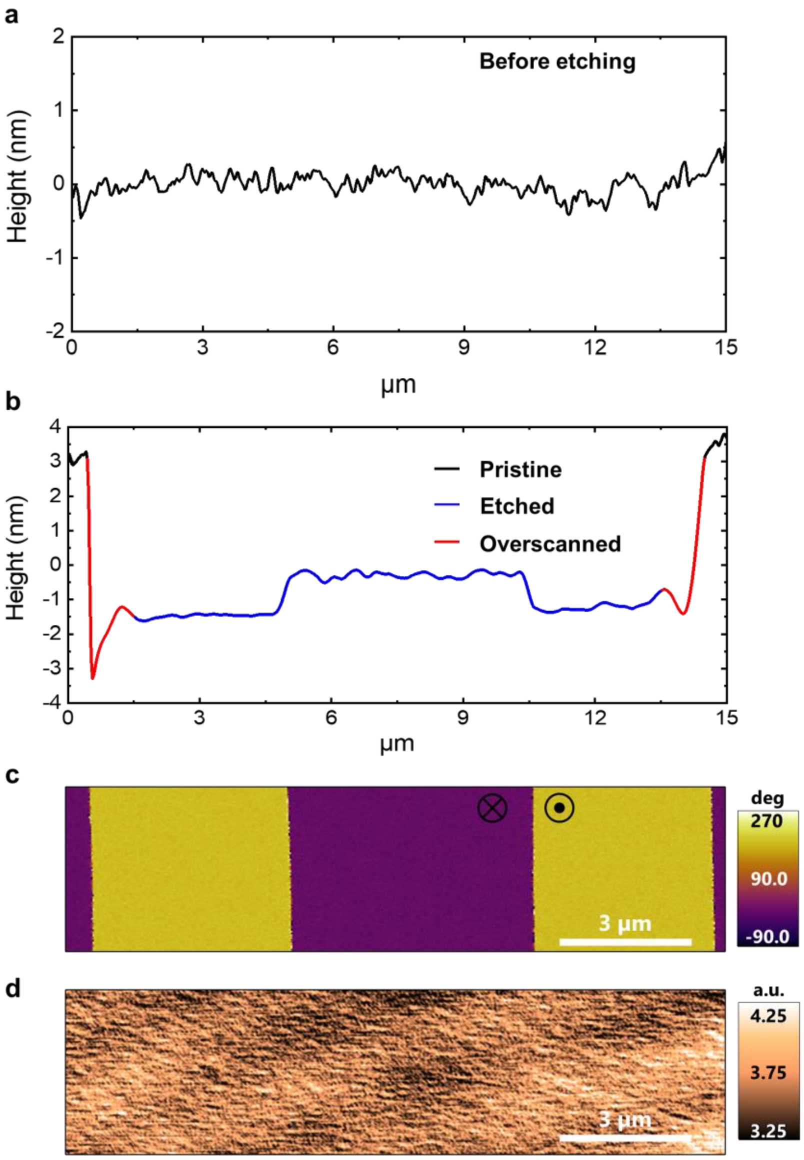

Fig. S3. Topography before and after mechanical grinding in Fig. 1 with pristine PFM phase and friction images. (a) Line profile along the direction perpendicular to the domain boundary before mechanical milling scans. **(b)** Line profiles after 50 milling scans: pristine (black), milled in the set region (blue) and milled in the over-scanning region (red). In the over-scanning region, the height is non-uniformly milled because of the distortion correction algorithm during the contact scanning. **(c)** PFM phase and **(d)** friction at approximately 300 nN before milling scans. The horizontal x-axis scales in the PFM and friction images correspond to the x-axis scales in (a) and (b).

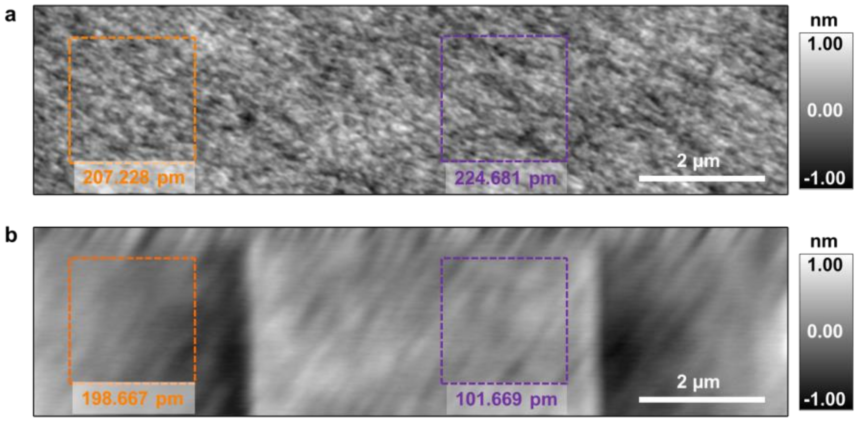

Fig. S4. Surface roughness before (a) and after (b) milling scans in Fig. 1. Orange represents the region acquired for roughness analysis in the up domains, while purple indicates the region for roughness analysis in the down domains. The roughness decreases after milling scans in both polarization domains.

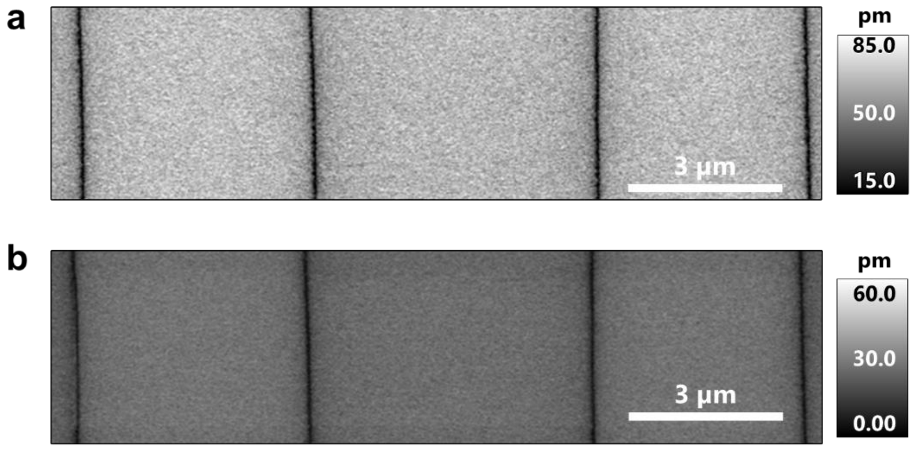

Fig. S5. PFM amplitude before and after the mechanical grinding shown in Fig. 1. PFM amplitude **(a)** before and **(b)** after mechanical milling scans. The amplitude scale changes because of the wear debris attachment to the tip during the scans.

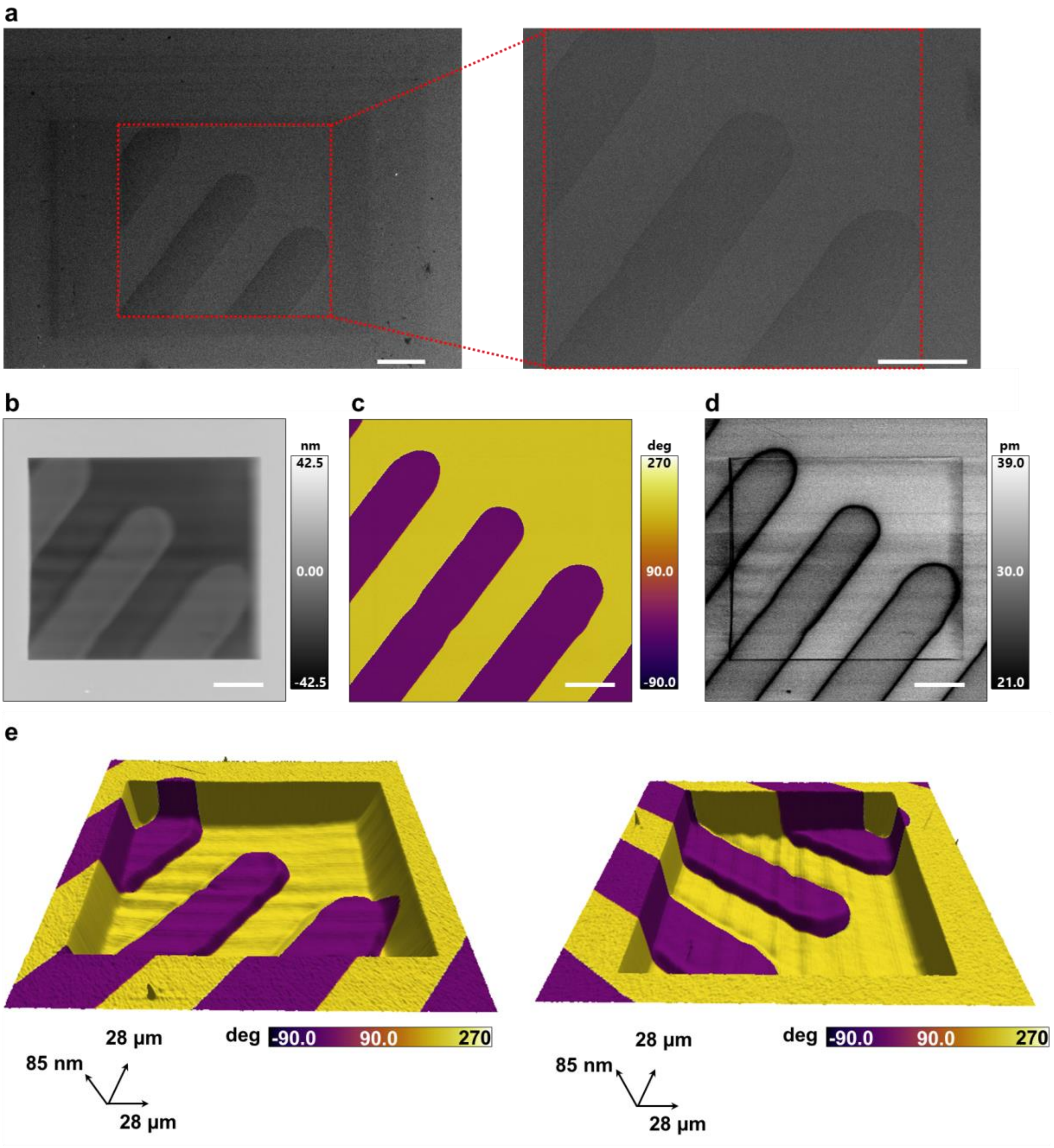

Fig. S6. Cross-validation of wear asymmetry using SEM. (a) SEM images, **(b)** Height, **(c)** PFM phase and **(d)** PFM amplitude after 50 milling scans. **(e)** 3D surface plots with a color scale indicating PFM phase. SEM imaging was performed 15 days after milling scans and PFM imaging. Comparing SEM images with PFM data, the SEM contrast originates from the height difference between up and down domains. Scale bars in (a–d) are 5 µm. The full-length scale along each respective axis is shown with an arrow in (e).

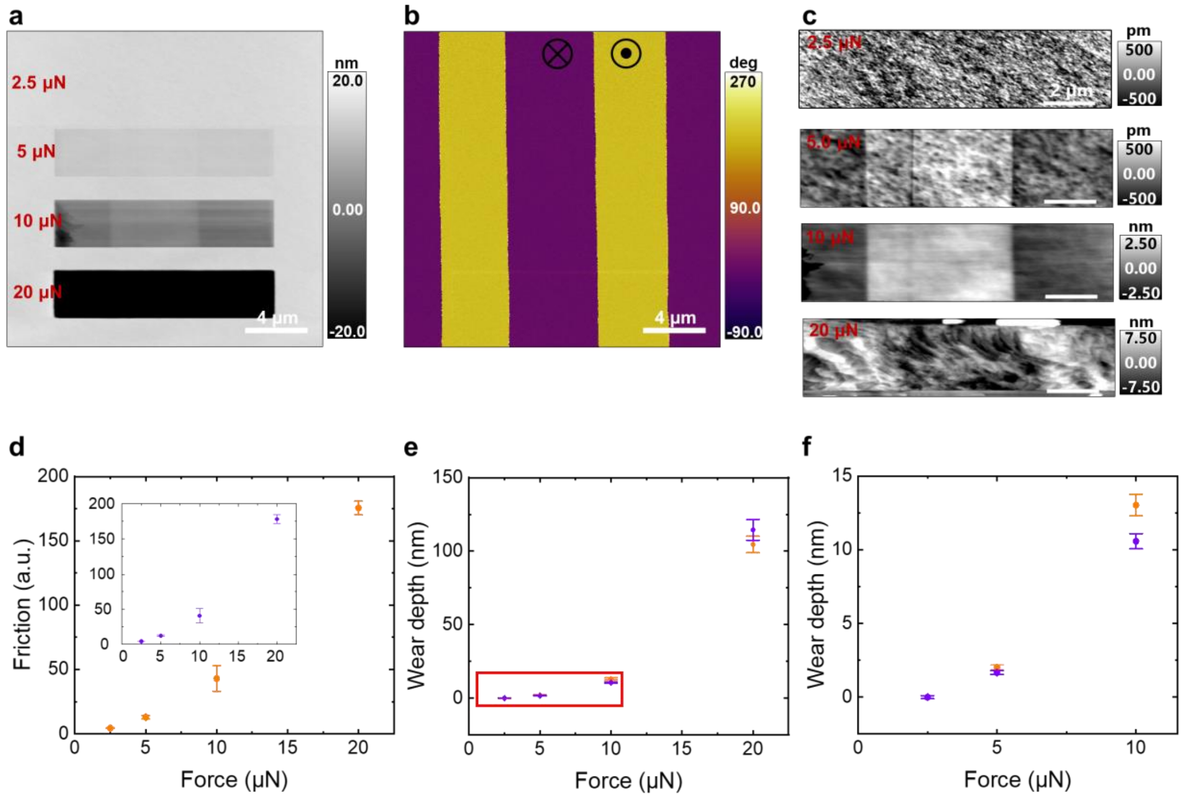

Fig. S7. **Contact force optimization of asymmetric friction and wear. (a)** Height image after ten milling scans with increasing loading forces in four different regions. **(b)** PFM phase image after ten milling scans. **(c)** Height in each region with different loading forces from 2.5 to 20 μN. **(d)** Friction in up domains as a function of loading force in each region. Inset shows friction vs. loading force in down domains. **(e)** Wear depth as a function of loading force in each region. **(f)** Wear depth as a function of loading force except for the failure case. Ten friction measurement points were averaged for each data point with error bars given by standard deviations.

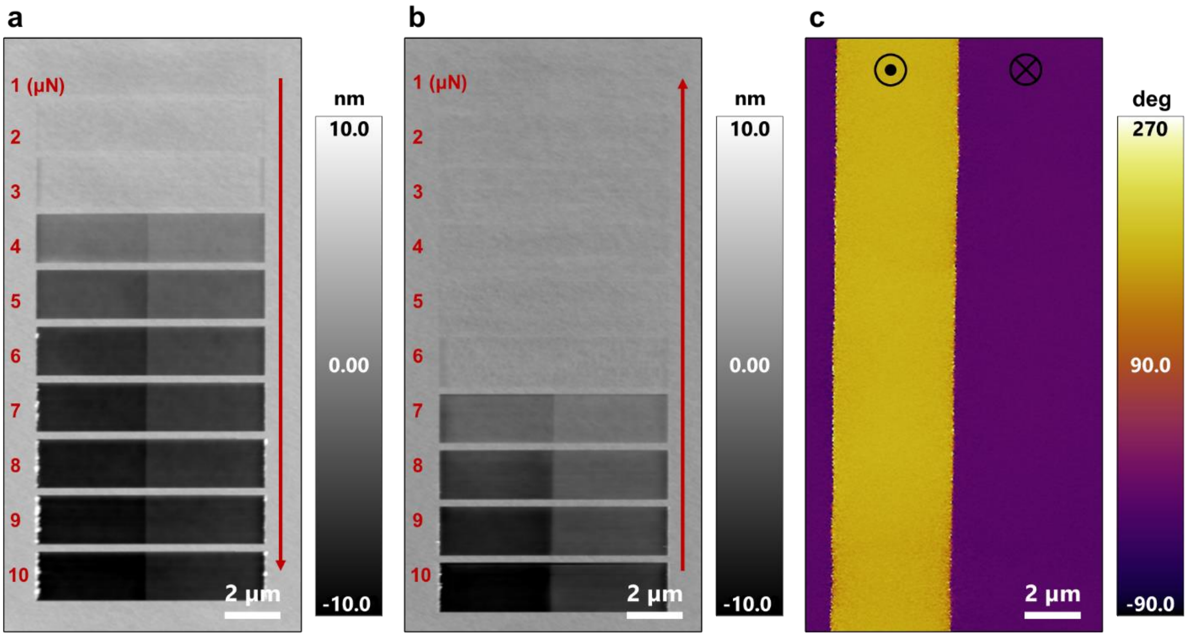

Fig. S8. **Effect of increasing and decreasing loading force on asymmetric tribology using the same probe.** Arrows indicate the order of milling scans and numbers in red show the loading forces applied in each region. Four milling scans were conducted in each region from a loading force of 1 μN to 10 μN. **(a)** Height after milling scans with increasing loading force from 1 μN to 10 μN. **(b)** Height after milling scans with decreasing loading force from 10 μN to 1 μN. Milling scans conducted in (b) were conducted after milling scans in (a) using the same probe. **(c)** PFM phase before mechanical milling scans in the same region as (b).

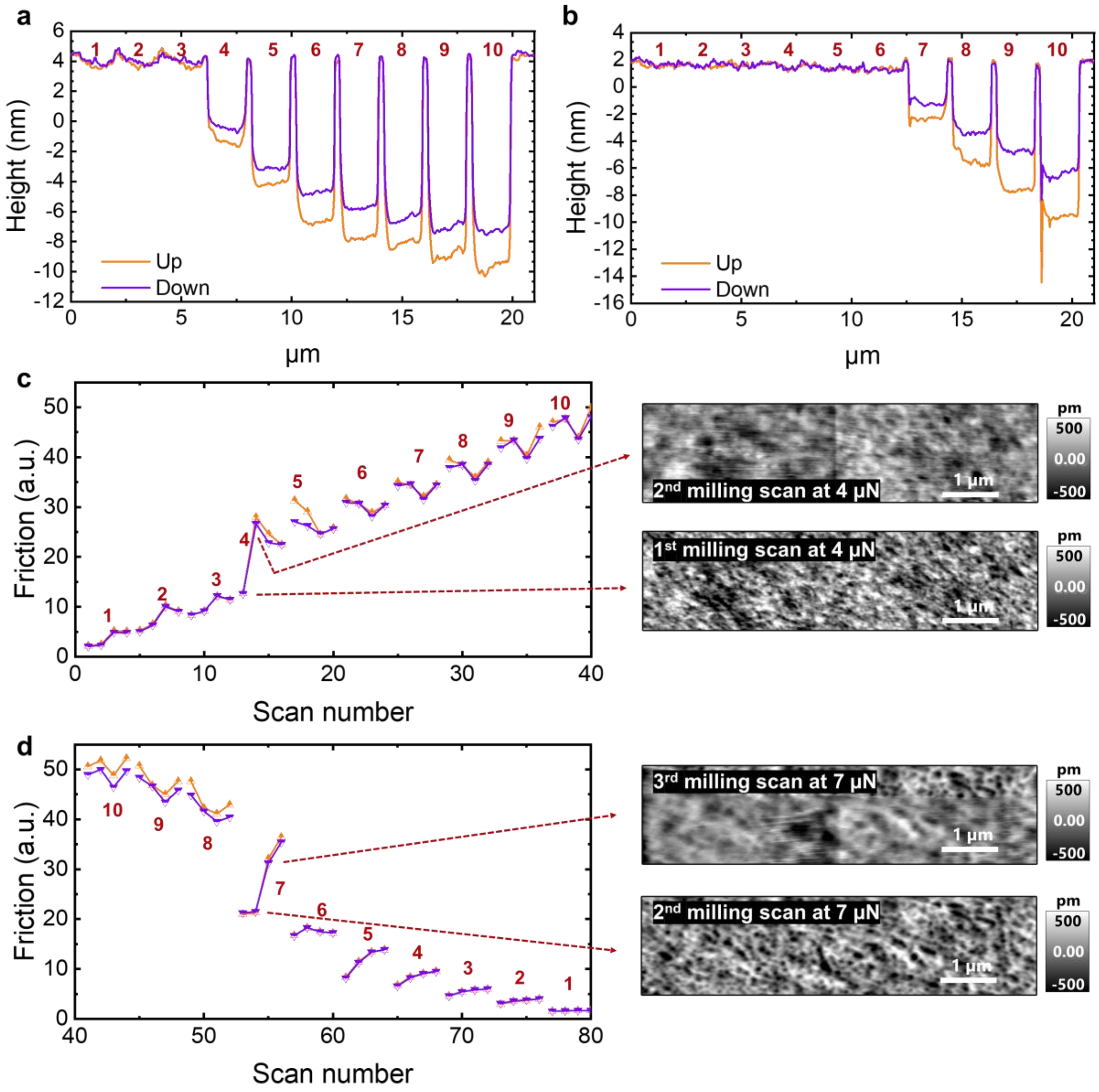

Fig. S9. **Friction and wear trend from the experiment in Fig. S8. (a and b)** Wear depth profiles of the up (orange) and down (purple) domains along the vertical (slow scan axis) direction in Figs. S8a and S8b, respectively. The numbers in red font indicate the loading force at each region. Four milling scans were performed in each region. **(c)** Friction vs. scan numbers for increasing loading force in Fig. S8a**.** Height during the 1st and 2nd milling scans at 4 μN, respectively. **(d)** Friction vs. scan numbers with decreasing loading force in Fig. S8b**.** Height during the 2nd and 3rd milling scans at 7 μN, respectively. Wear asymmetry is observed with the jump of friction signals seen in (c) and (d).

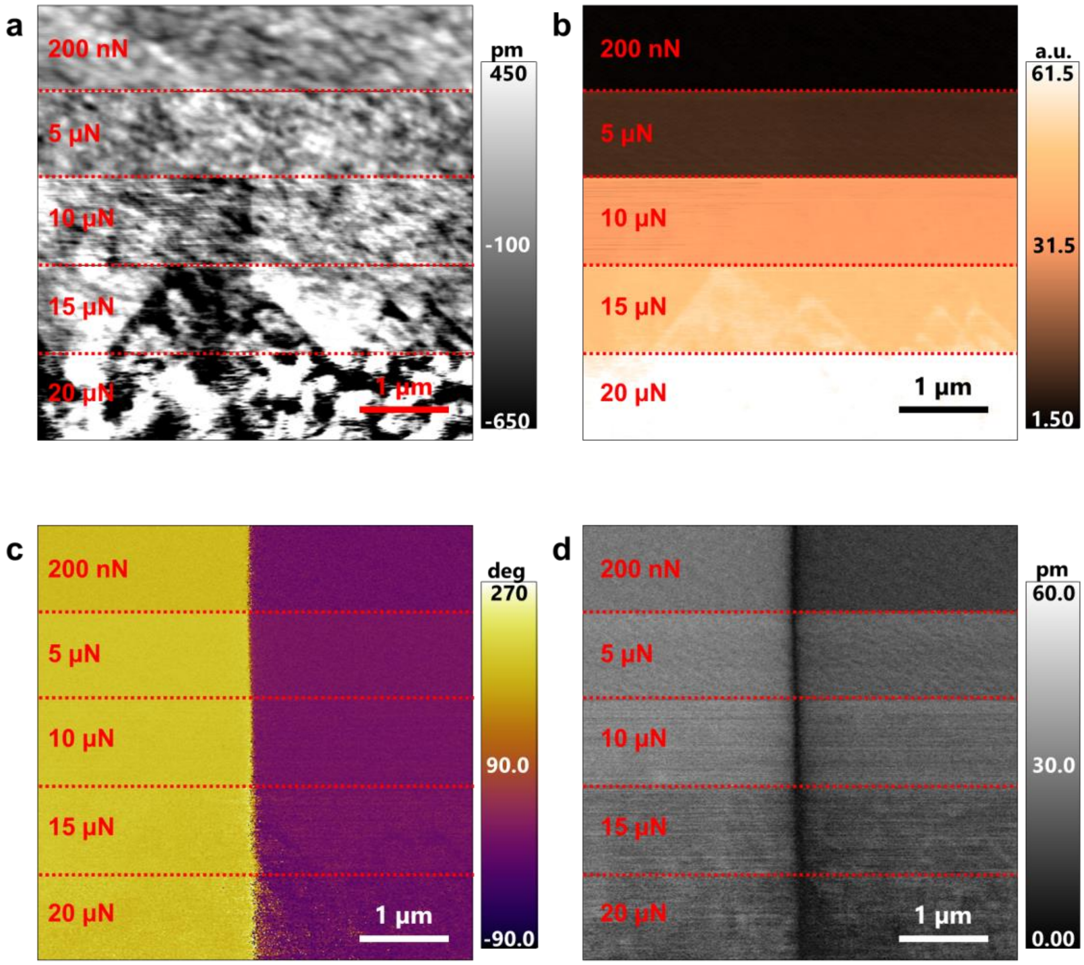

Fig. S10. Simultaneous PFM imaging of PPLN with increasing loading force from 200 nN to 20 µN showing no transient domain switching during the application of high strain gradient. (a) Topography, **(b)** friction, **(c)** PFM phase, and **(d)** PFM amplitude during the PFM scanning with increasing loading force. At high loading forces such as 15 µN and 20 µN, flexoelectric switching is not observed even with material fractures during the scan.

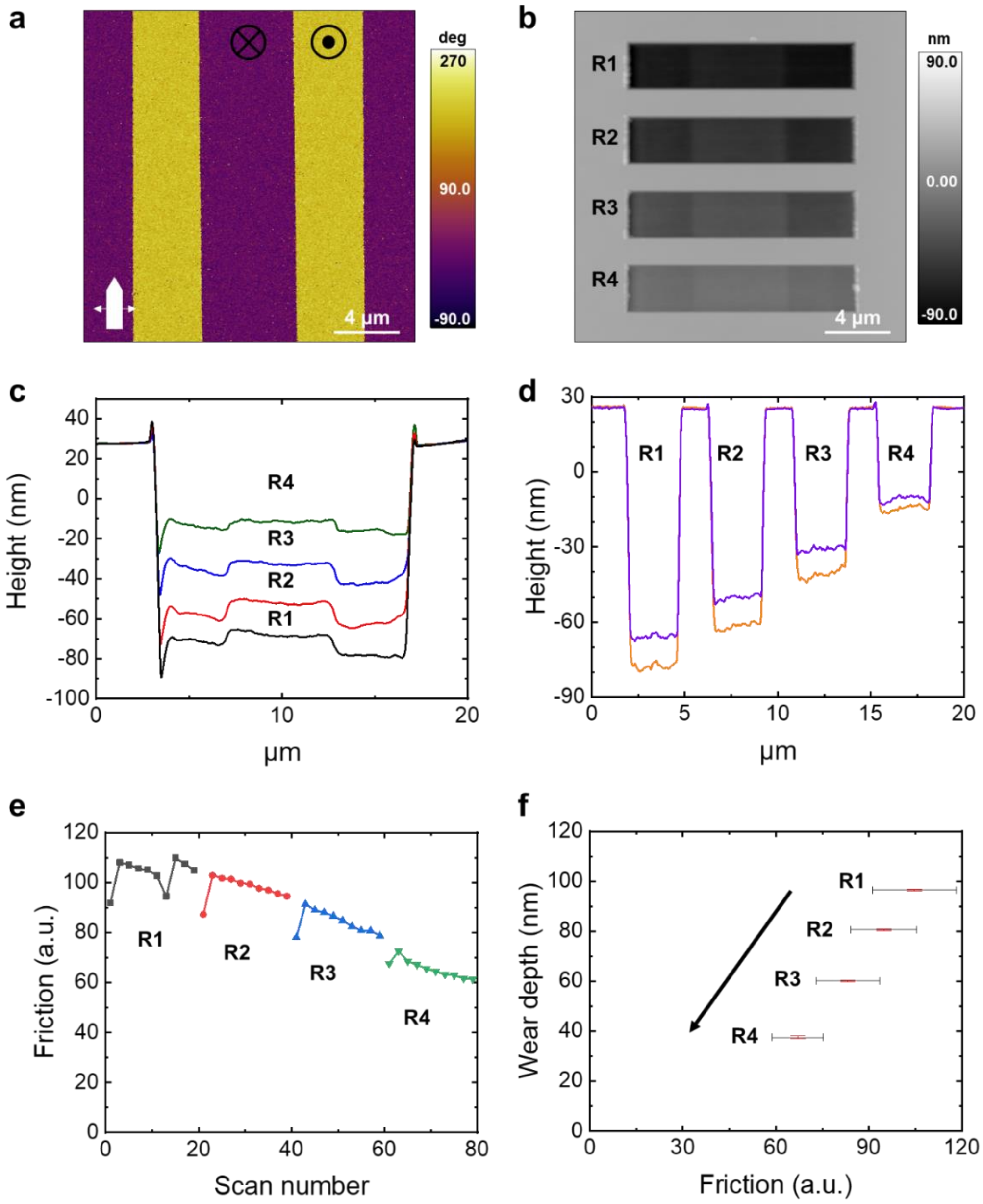

Fig. S11. Friction and wear degradation with continuous scans. (a) PFM phase image before milling scans, **(b)** height image after milling on four different regions from region 1 (R1) to region 4 (R4) with the same polarity. A loading force of 5 μN was applied at a scan rate of 1.95 Hz in each scan, and 10 milling scans were performed in each region. **(c)** Horizontal line profile along the milled regions. **(d)** Vertical line profiles along the milled height indicate the wear rate difference between the up (orange) and down domains (purple). **(e)** Friction signal degradation from R1 to R4. **(f)** Correlation between average friction and wear with standard deviations. All scans were measured at 20°C and 20% relative humidity. 20 measurement points were averaged for each data point with error bars given by standard deviations.

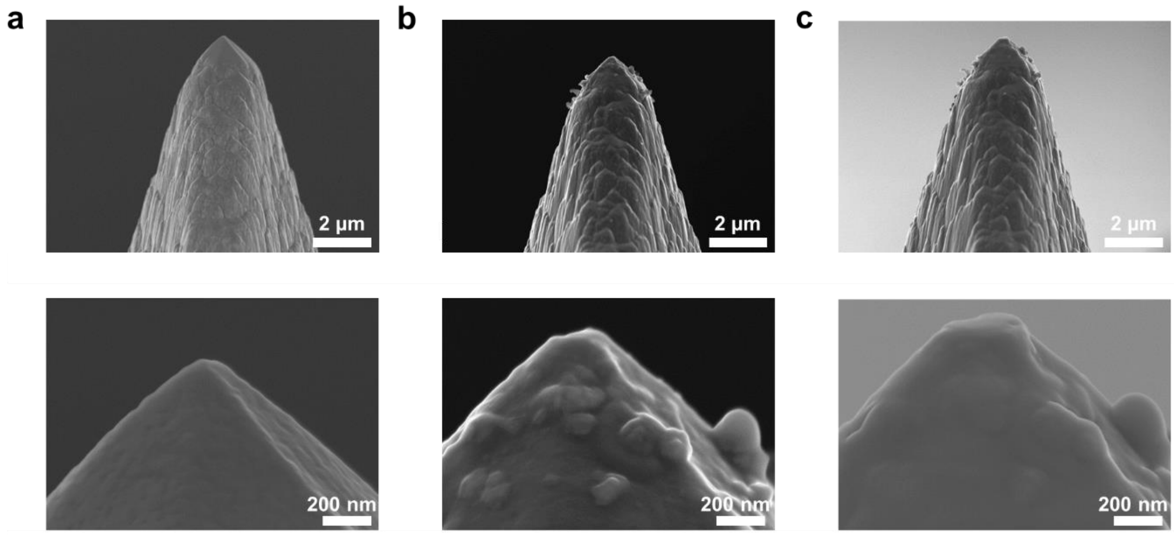

Fig. S12. SEM images of the diamond probe before and after milling. (a) Pristine, **(b)** after 10 millings, **(c)** after 50 millings at a loading force of 10 μN and a scan rate of 4.88 Hz on 20 μm × 20 μm of PPLN surface. The attachment of wear debris during continuous millings is evident. The end of the probe has a dull shape covered by debris as compared to the initial sharp cone shape. The debris coverage on the diamond probe is correlated with the degradation of the friction and wear depth during continuous milling.

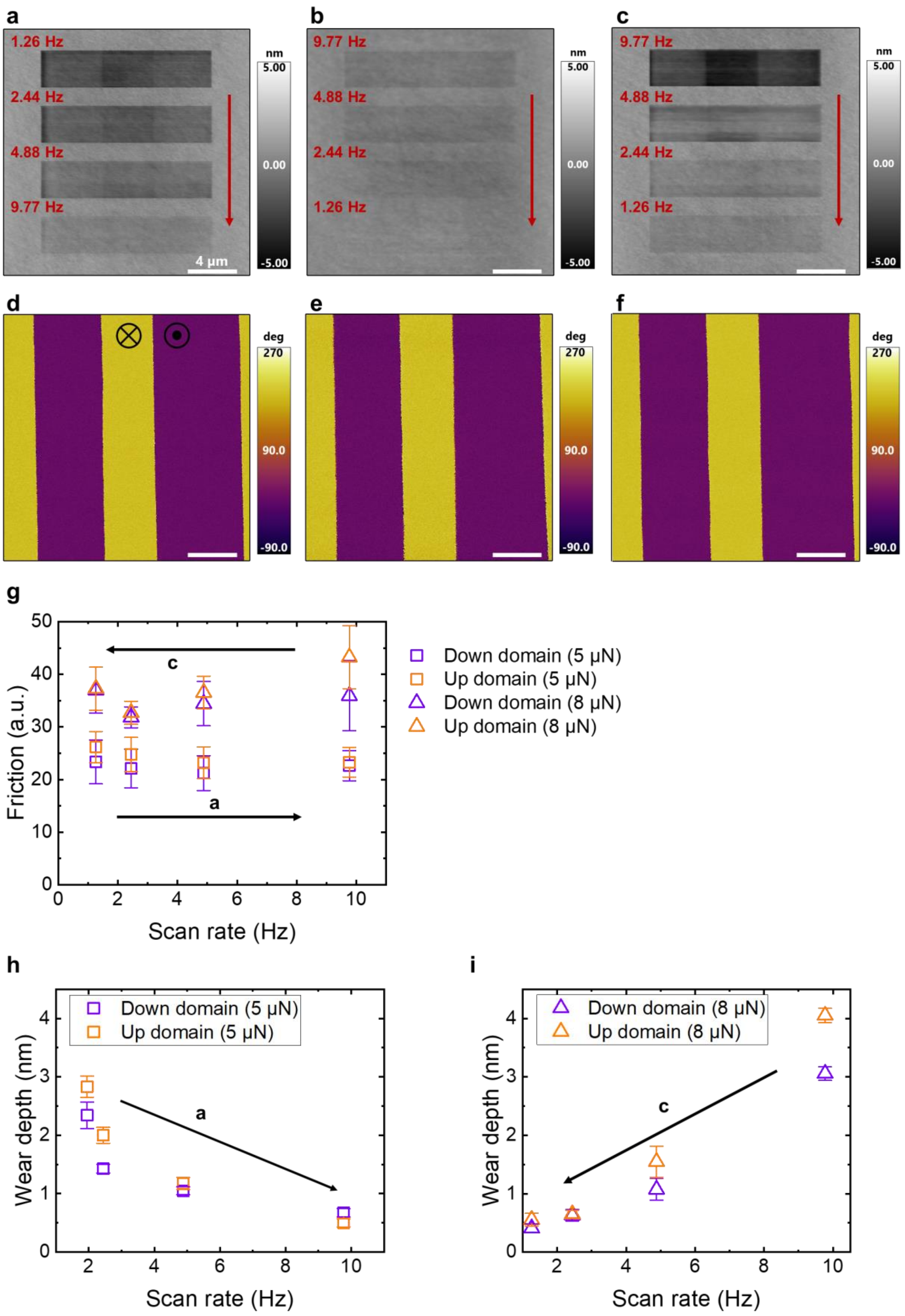

Fig. S13. Effect of scan rate on asymmetric tribology. Red arrows indicate the order of milling scans and numbers in red font show the scan rate applied in each region. **(a)** Height

image after ten milling scans with four different scan rates at a loading force of 5 μN. **(b)** Height image after ten milling scans with four different scan rates at 5 μN after milling scans in (a) using the same probe. **(c)** Height image after ten milling scans with four different scan rates at 8 μN after milling scans in (c) using the same probe. Red arrows indicate the scan order. **(d–f)** PFM phase images of (a–c), respectively. **(g)** Friction vs. scan rate in (a) and (c). **(h)** Wear depth depending on the scan rate in (a). **(i)** Wear depth depending on scan rate in (c). Scan order is indicated with arrows in (g–i). 30 measurement points were averaged for each data point with error bars given by standard deviations.

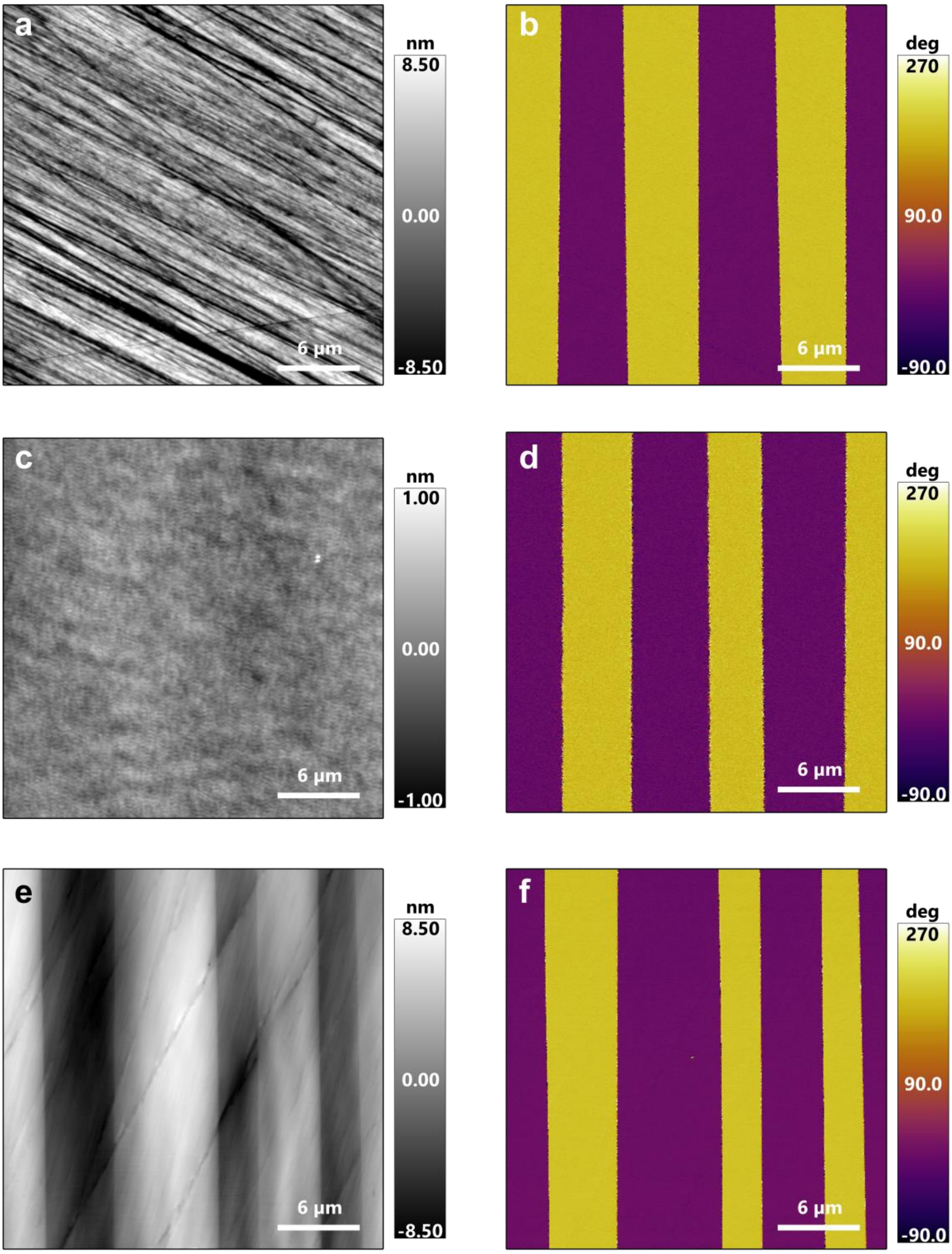

Fig. S14. Control experiments of scalable large-area milling of PPLN. (a) Height and **(b)** PFM phase after mechanical grinding using diamond lapping films. **(c)** Height and **(d)** PFM phase after dipping in colloidal silica solution for 12 h. **(e)** Height and **(f)** PFM phase after polishing using silica nanoparticles for 3 min.

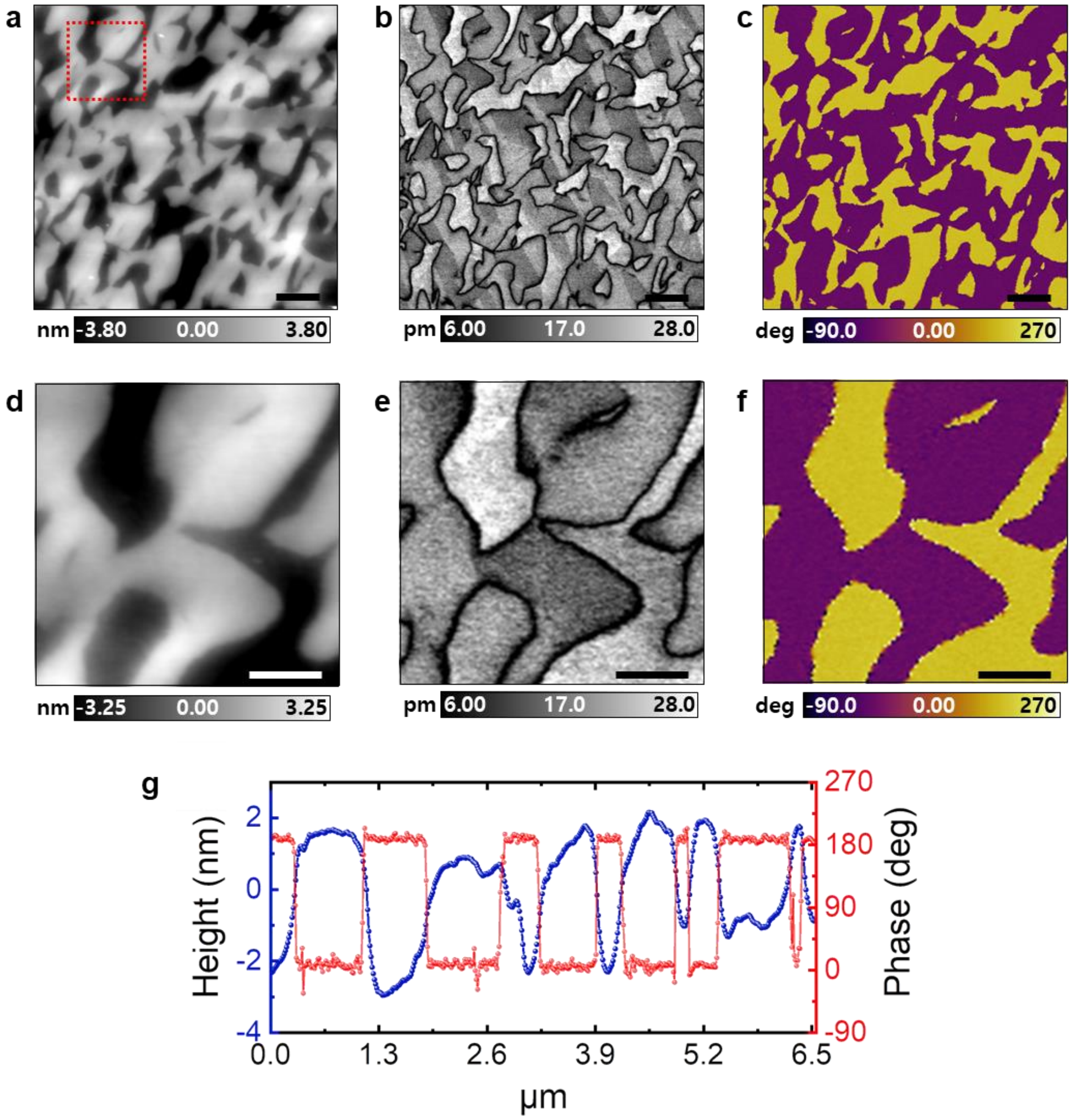

Fig. S15. Scalable polarization-derived lithography of PZN-PT single crystals. (a) Height, **(b and c)** vertical PFM amplitude and phase after polishing using silica nanoparticles for 20 min. **(d)** Height, **(e)** vertical PFM amplitude and **(f)** phase in the red box in (a). **(g)** Line profiles of height and PFM phase obtained from (a) and (b). Scale bars are 1 μm for (a–c) and 400 nm for (d–f).

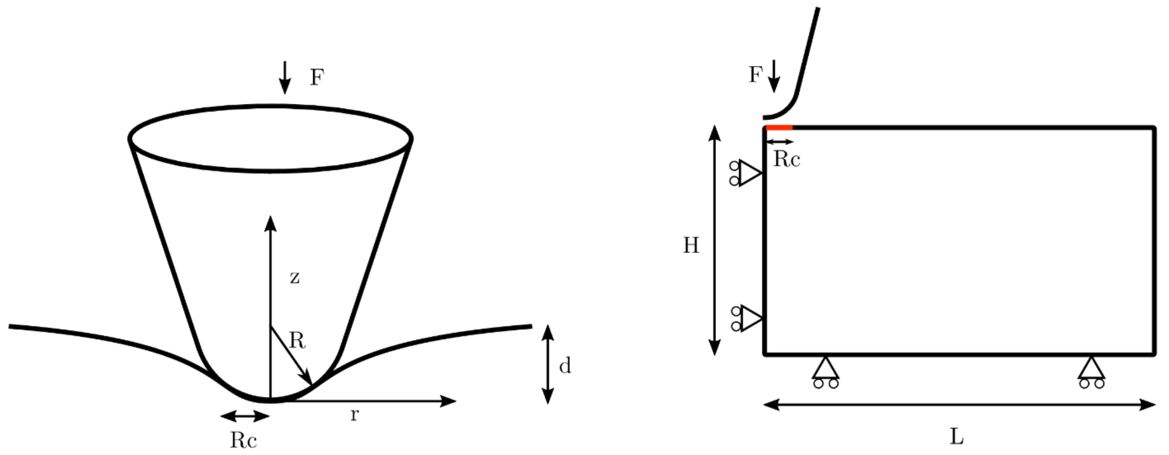

Fig. S16. Geometrical parameters of the spherical indenter (left) and axisymmetric problem statement with mechanical boundary conditions (right). A rectangle of dimensions H and L is simulated, the left side of the rectangle is clamped horizontally and the bottom side is clamped vertically.

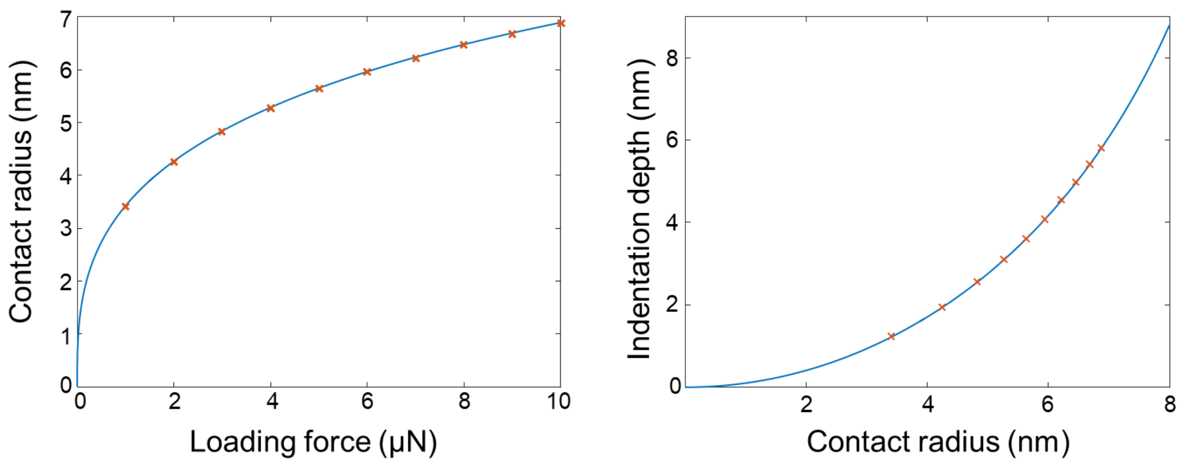

Fig. S17. Validation of the contact model against the analytical model for linear elasticity (vanishing $\boldsymbol{e}, \boldsymbol{\mu}, \boldsymbol{\kappa}$ and $\boldsymbol{h}$)[7]. Contact radius as a function of applied force (left) and indentation depth as a function of contact radius (right).

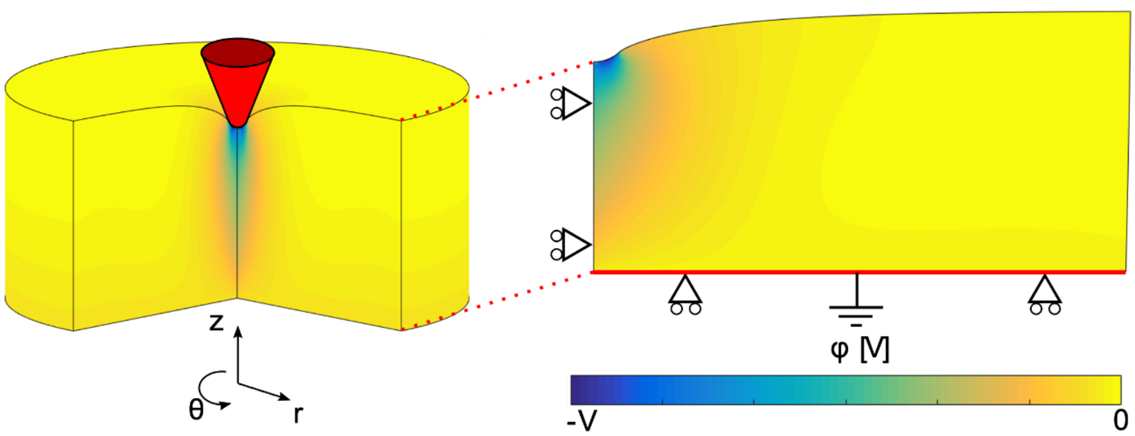

Fig. S18. Schematic of the axisymmetric three-dimensional model depicting the electric potential distribution upon indentation using a spherical AFM probe. In the two-dimensional axisymmetric model, the horizontal length is 40 nm and the vertical height is 20 nm**.**

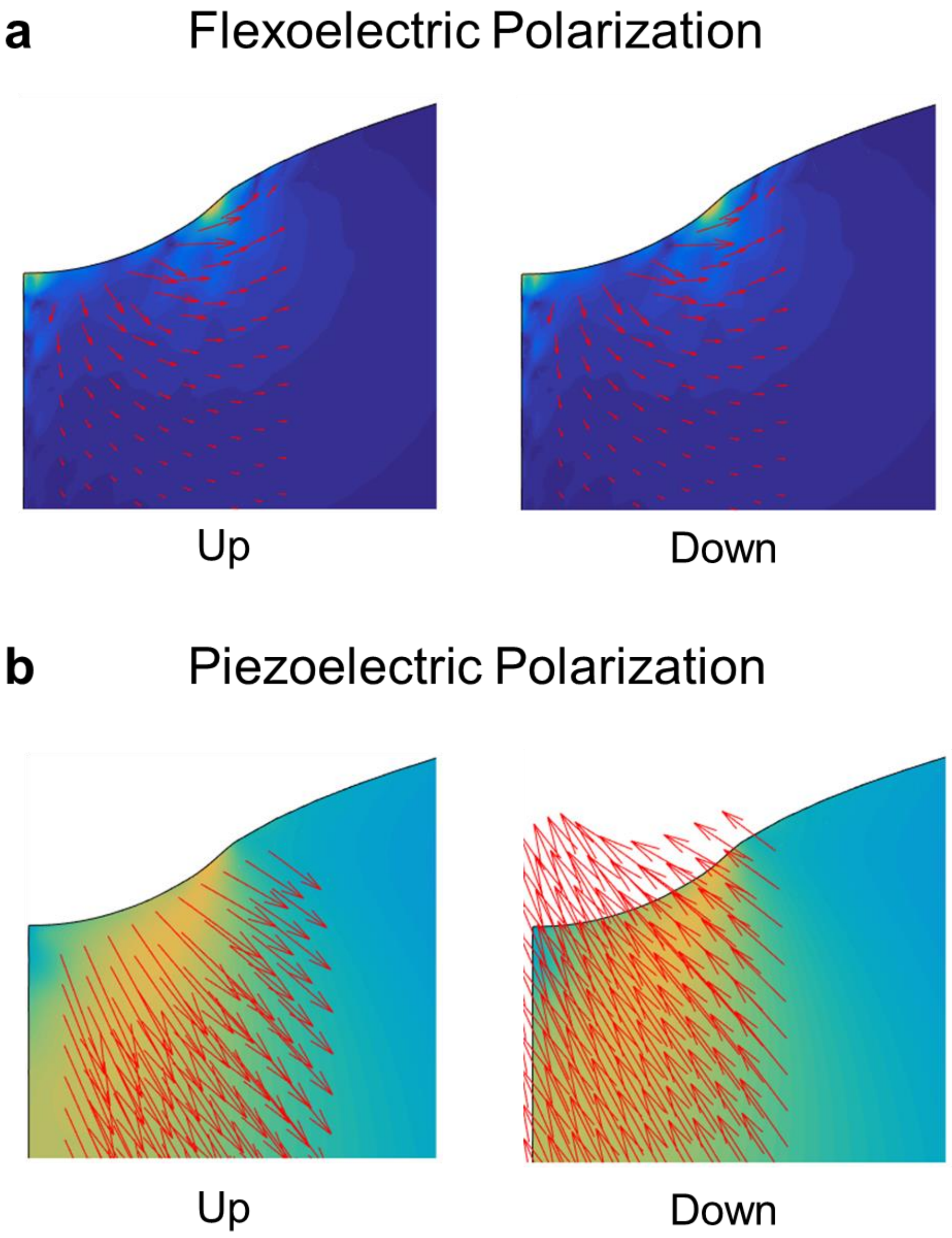

Fig. S19. **Polarization fields upon indentation induced by flexoelectricity and piezoelectricity for up and down ferroelectric domains, for $\boldsymbol{f = 10\ V}$. (a)** Flexoelectrically induced polarization for the up and down domains. **(b)** Piezoelectrically induced polarization for the up and down domains. The polarization plotted here is relative to the remanent polarization. The arrows represent the induced polarization at the starting point of the polarization vector.

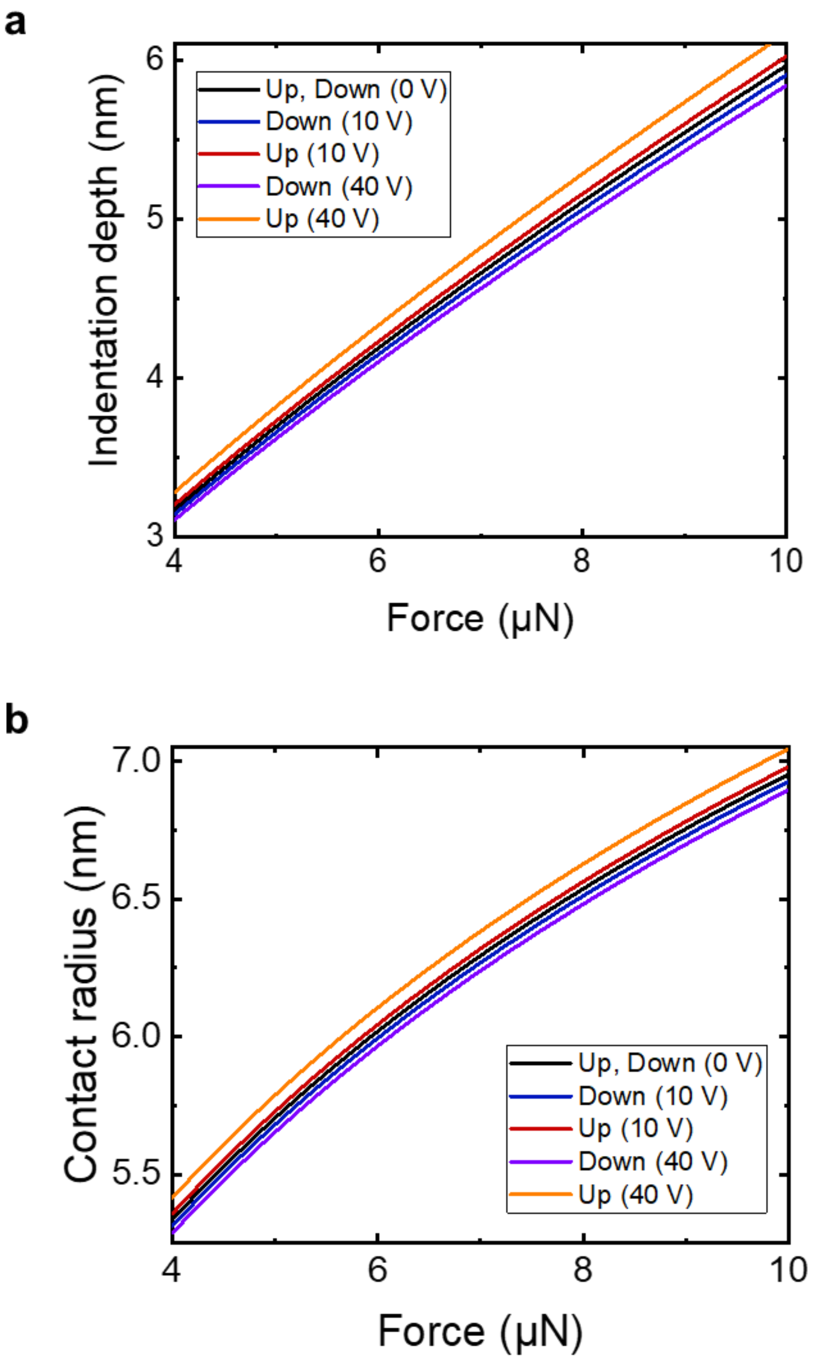

Fig. S20. The effect of flexoelectricity on indentation depth and contact radius in up and down domains. (a) Indentation depth and **(b)** contact radius as functions of applied force for up and down polarized domains for different values of the flexocoupling coefficient: $f = 0\ V$, $f = 10\ V$ and $f = 40\ V$. The observed asymmetry between up and down domains is stronger for larger f and vanishes in the absence of flexoelectricity ($f = 0\ V$). Flexocoupling coefficients are based on values in Refs. [13–15].

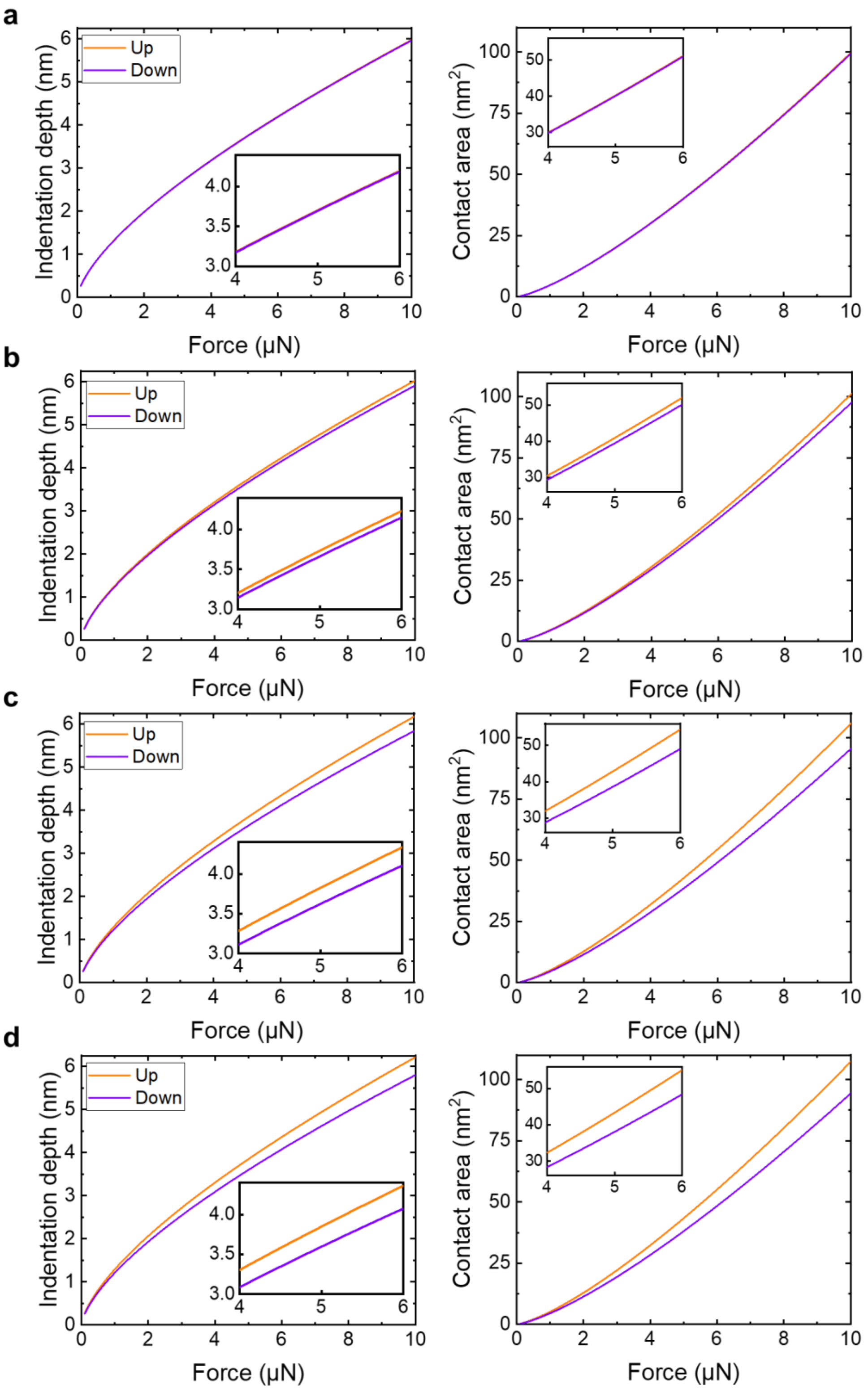

Fig. S21. Asymmetric indentation depth and contact area of ferroelectric up (yellow) and down (purple) domains with different flexocoupling coefficients. Indentation depth and contact area **(a)** $f = 1\ V$, **(b)** $f = 10\ V$, **(c)** $f = 40\ V$ and **(d)** $f = 54\ V$. Flexocoupling coefficients in (c) and (d) are based on experimental values in Ref. [15].

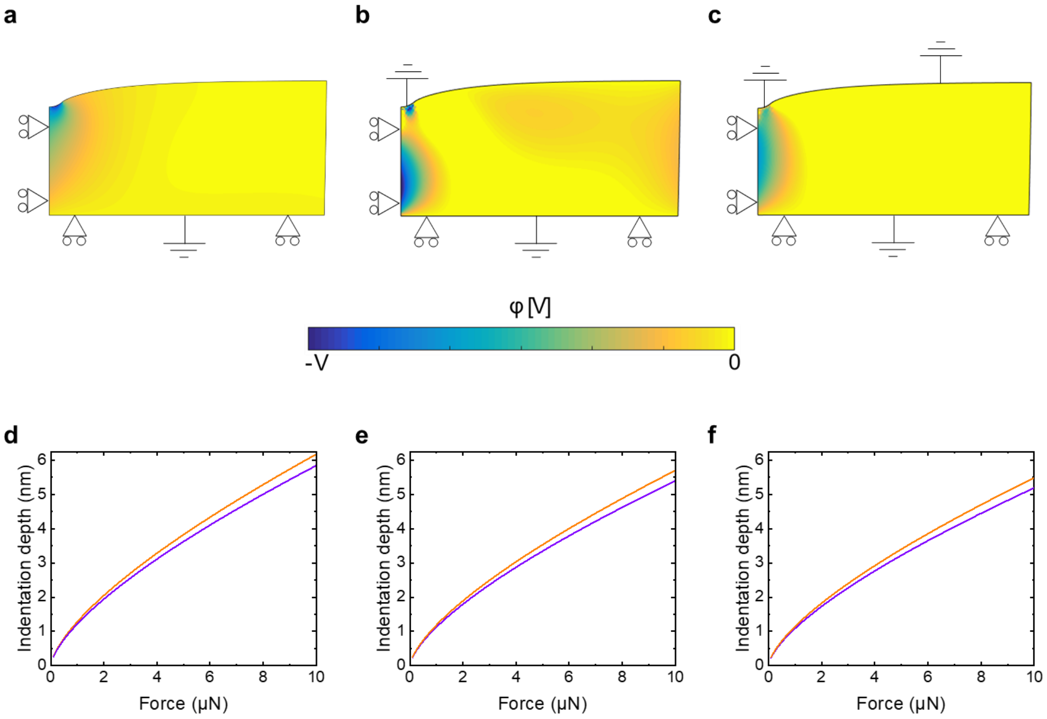

Fig. S22. Electric potential distribution and indentation depth depending on electric ground condition. (a–c) Electric potential distributions upon indentation using the spherical AFM probe with different grounding conditions. Electric potential distribution considering the bottom of the sample is as ground (a). Electric potential distribution considering the bottom of the sample and the contact zone are as ground (b). Electric potential distribution considering the bottom of the sample, the surface of the sample, and the contact zone are as ground (c). **(d–f)** Simulated indentation depth for (a–c), respectively. Orange is used for the up domains and purple is used for the down domains.

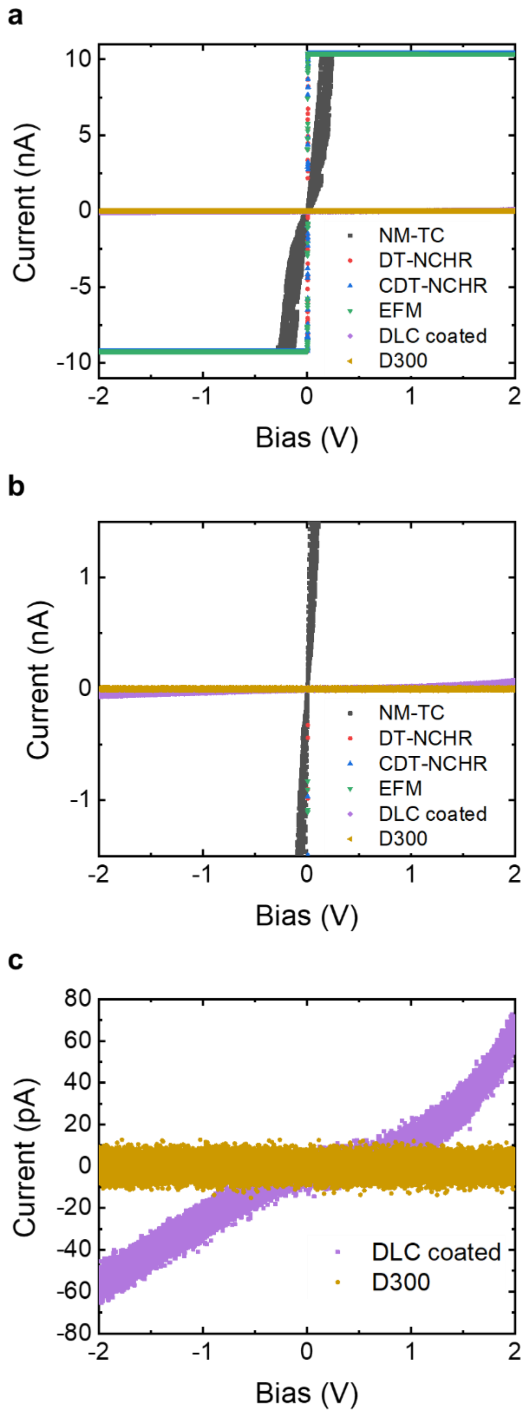

Fig. S23. ***I-V* curve measurements on highly ordered pyrolytic graphite (HOPG) using six different probe types. (a)** *I-V* curves on HOPG with six different probes. **(b)** Magnified *I-V* curves from (a) and **(c)** *I-V* curves on HOPG using the DLC-coated probe (weakly conductive) and the D300 probe (non-conductive).

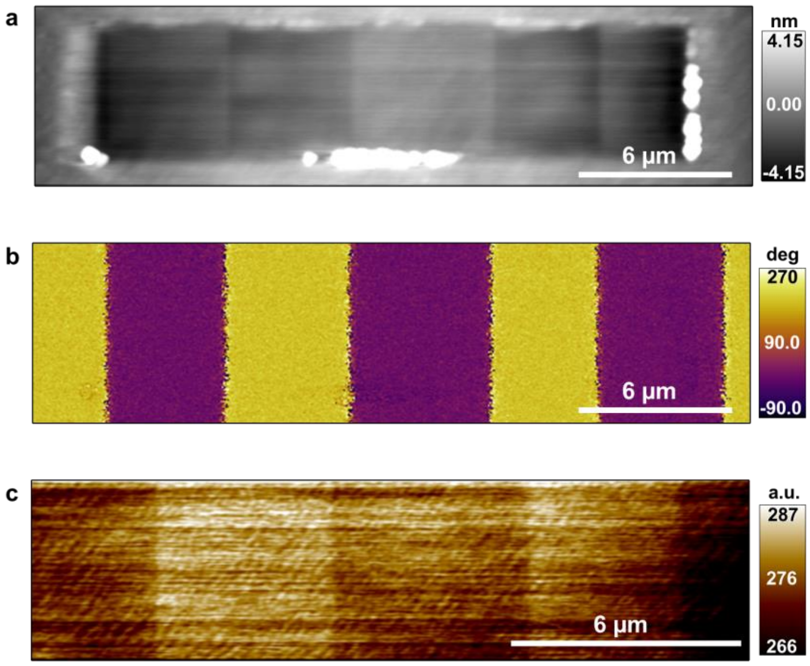

Fig. S24. Tribological asymmetry with a metal-coated Si probe. (a) Topography and **(b)** PFM phase after the multiple milling scans using a Spark 350-Pt probe (NuNano). **(c)** Friction image during the milling scan showing higher friction in the up domains and lower friction in the down domains.

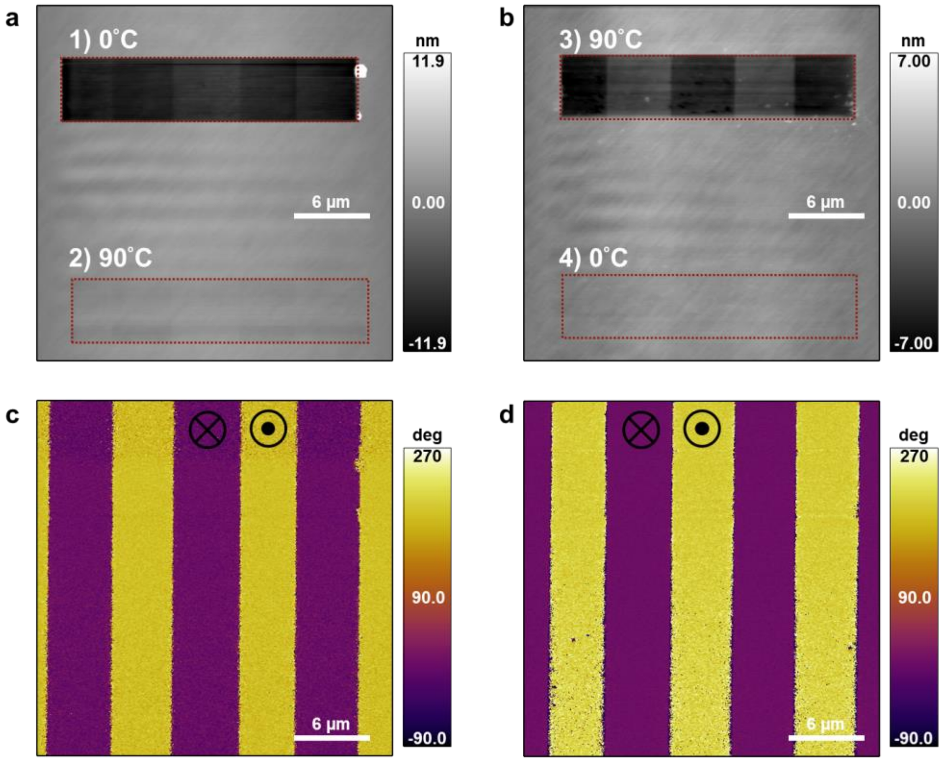

Fig. S25. Temperature-dependent milling experiment on PPLN. (a) Height after milling scans from 0˚C to 90˚C including the worn regions at a loading force of 10 µN. **(b)** Height after milling scans including the worn regions from 90˚C to 0˚C at a loading force of 11.5 µN using the same probe used in (a). In the milling experiment, a single probe (NM-TC) was used and the loading force was slightly increased from 10 to 11.5 µN for comparison under optimized conditions. **(c)** PFM phase after milling scans including the worn regions from 0˚C to 90˚C. **(d)** PFM phase after milling scans including the worn regions from 90˚C to 0˚C.

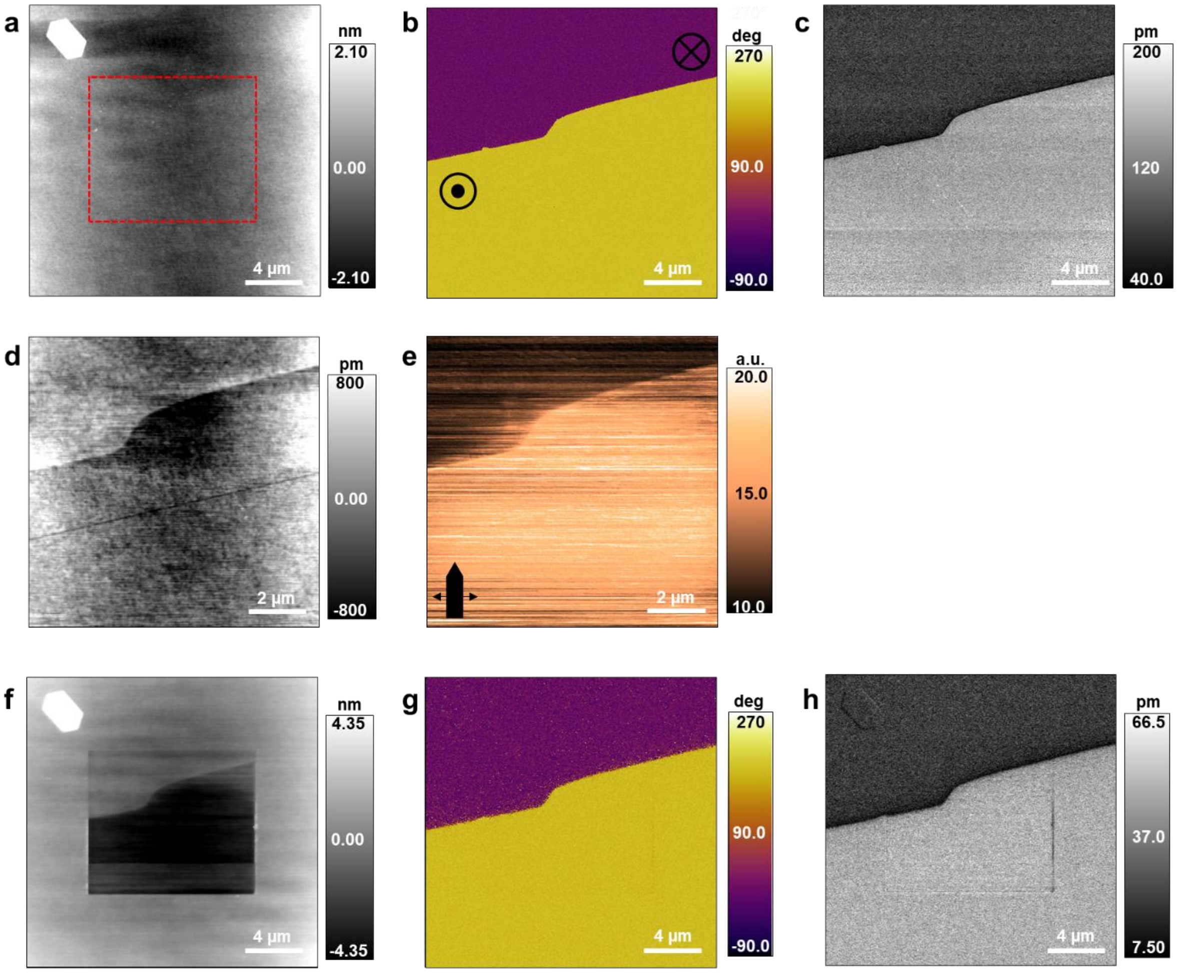

Fig. S26. Asymmetric friction and wear of stoichiometric $LiNbO_3$ single crystal. (a) Height, **(b)** PFM phase and **(c)** PFM amplitude of pristine surface of stoichiometric $LiNbO_3$. **(d)** Height, **(e)** friction during the milling scan. **(f)** Height, **(g)** PFM phase and **(h)** PFM amplitude after ten milling scans at a loading force of 5 μN and a scan rate of 1.95 Hz. All scans were measured at 20°C and 20% relative humidity.

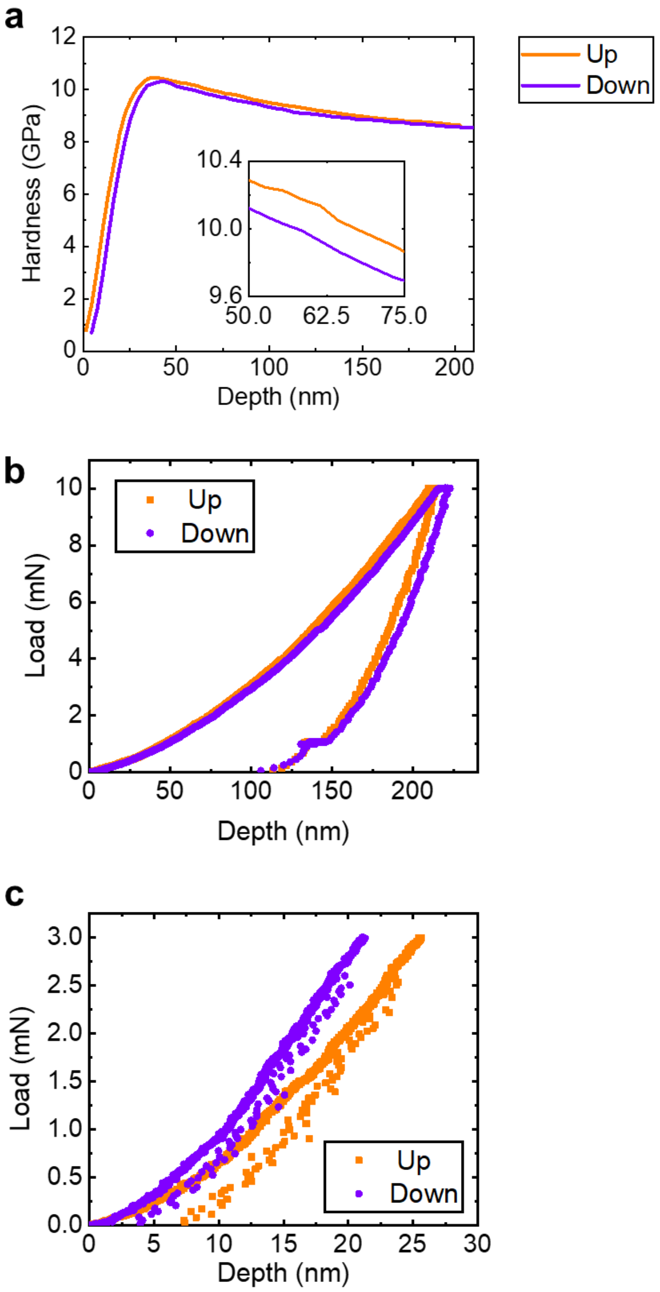

Fig. S27. Nanoindentation of stoichiometric $LiNbO_3$ single crystals. (a) Hardness and **(b)** load-depth curve of ferroelectric up and down crystals from the Berkovich nanoindentation measurements. The maximum load is 10 mN. Ferroelectric up crystal shows higher hardness, so lower indentation depth than down crystal. **(c)** Loading and unloading load-depth curve using a spherical nanoindenter in ferroelectric up and down crystals. Ferroelectric up crystal shows a higher indentation depth than the down crystal. The maximum load is 3 mN. The average values of indentation depth in each case are shown in Table S3.

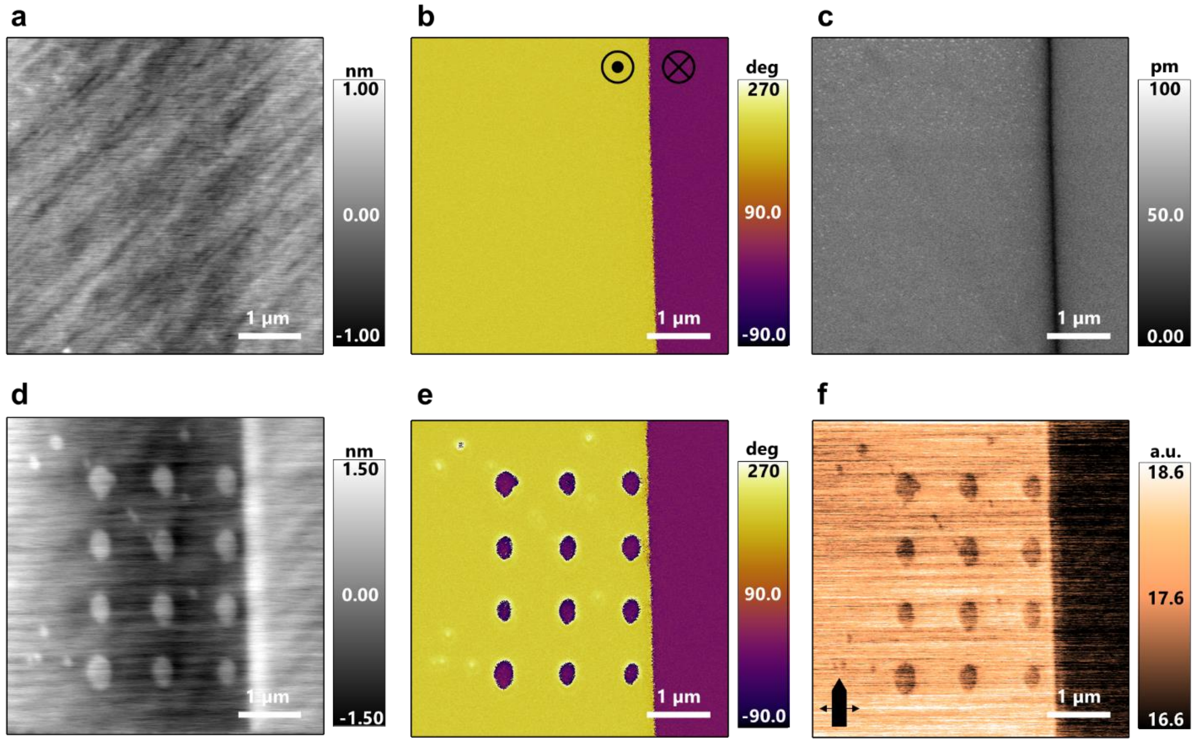

Fig. S28. Switchable tribological asymmetry in PPLN. (a) Height, **(b)** PFM phase and **(c)** PFM amplitude of pristine PPLN. **(d)** Height and **(e)** PFM phase after switching 3 × 4 arrays of down domains and ten milling scans at optimized condition. **(f)** Friction during the fifth milling scan. The friction of switched domains is lower than that of up domains, and the resulting topography is also higher in the switched domains. All scans were measured at 20°C and 20% relative humidity.

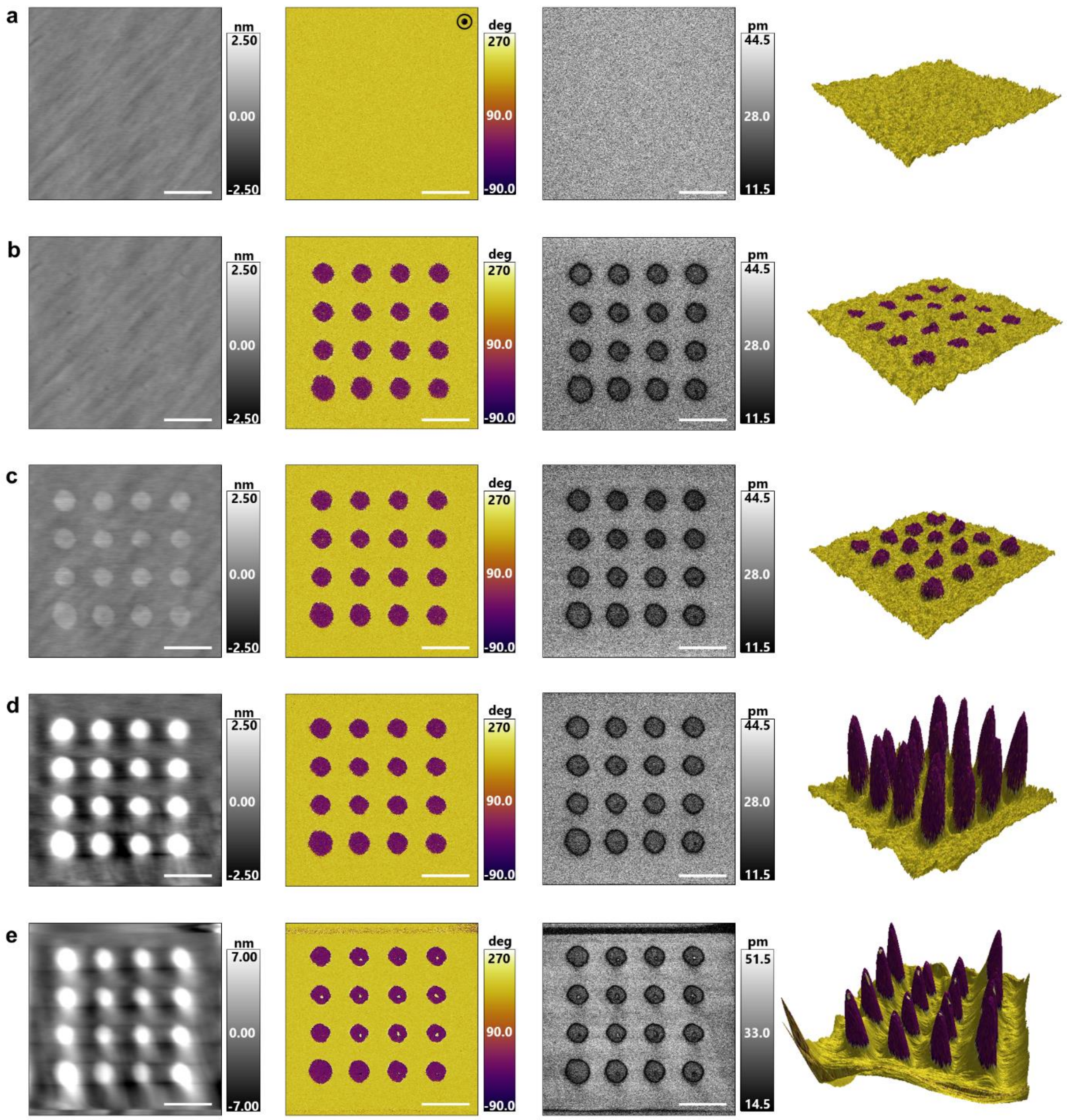

Fig. S29. **Fabrication of nanopillars in Fig. 3.** Height, PFM phase, PFM amplitude, and 3D image of height superimposed by PFM phase of **(a)** pristine up domain of PPLN, **(b)** after writing domains by electrical bias through the diamond probe, **(c)** after two millings, **(d)** after twenty millings, and **(e)** after sixty millings. Images in (e) show the existence of up domain at the core of the pillar, maybe because of the insufficient bias to switch the crystal. The z-scale of the 3D image is identical to that of the 2D height image. All scans were measured at 20°C and 20% relative humidity.

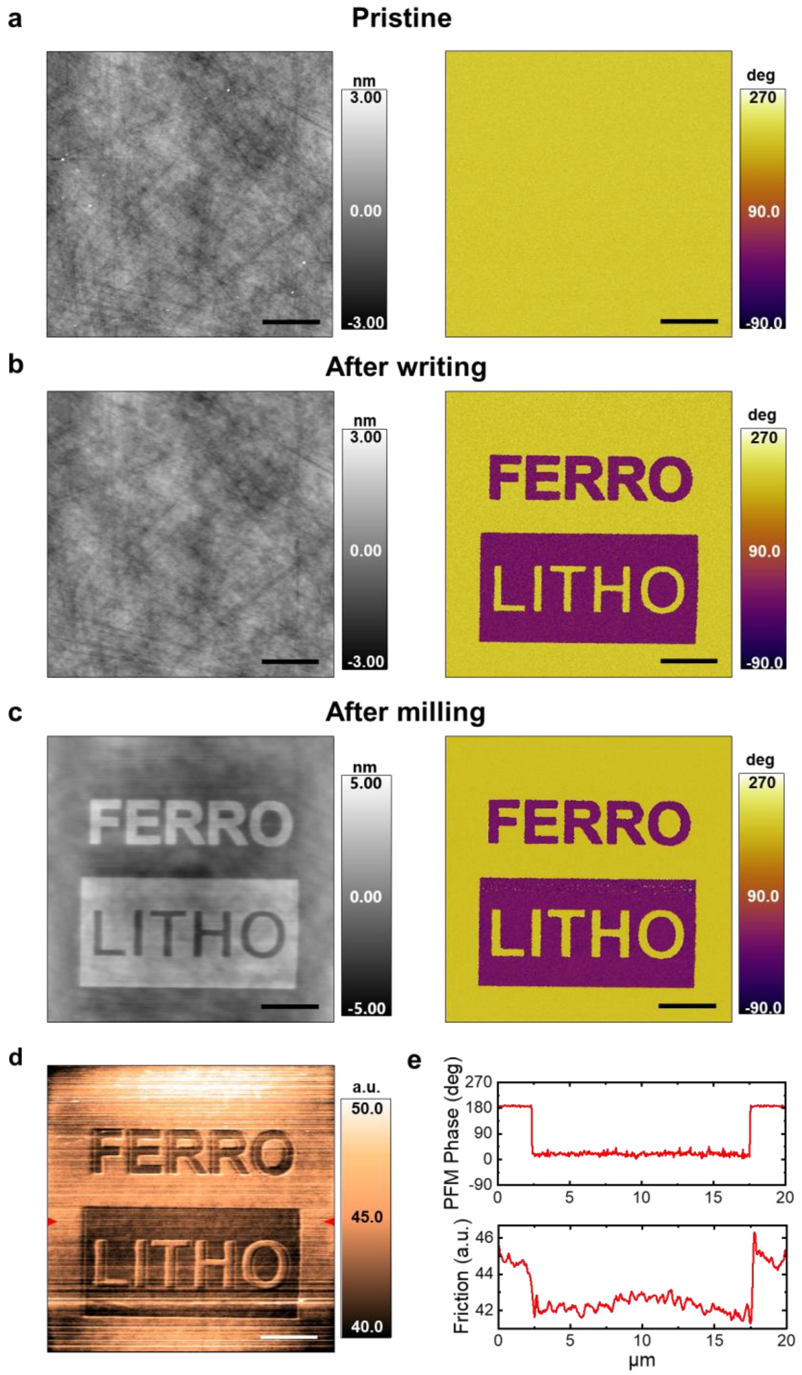

Fig. S30. Nanostructuring using switchable wear asymmetry of ferroelectric $LiNbO_3$ thin film. (a) Height and PFM phase of pristine $LiNbO_3$ thin film. **(b)** Height and PFM phase after the domain switching of "FERRO" and rectangular background of "LITHO" with the down domains. **(c)** Height and PFM phase after multiple milling scans. **(d)** Friction during the milling scan showing higher friction in the up domains and lower friction in the down domains. **(e)** Line profiles of friction and PFM phase along the red markers in (d). The line profile of PFM phase was obtained from (c). All scans were measured at 20°C and 20% relative humidity. Scale bars are 4 μm.

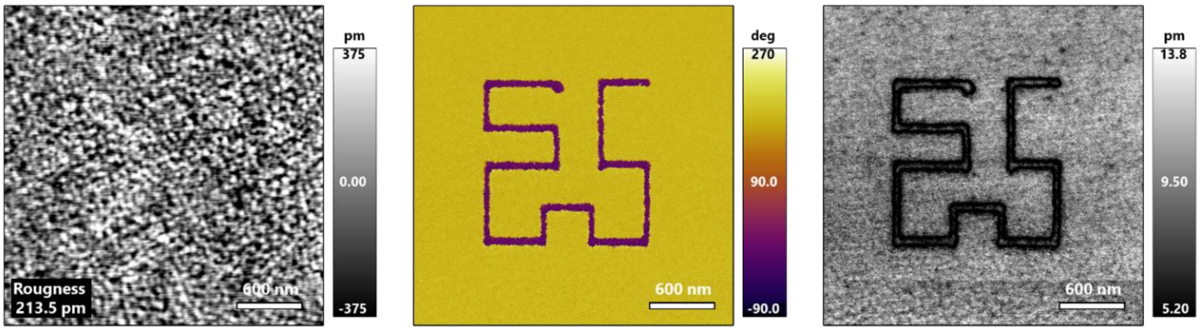

Fig. S31. Height (left), PFM phase (middle) and PFM amplitude (right) images obtained after the electrical switching of up domains to down domains by applying 5 V to the AFM tip.

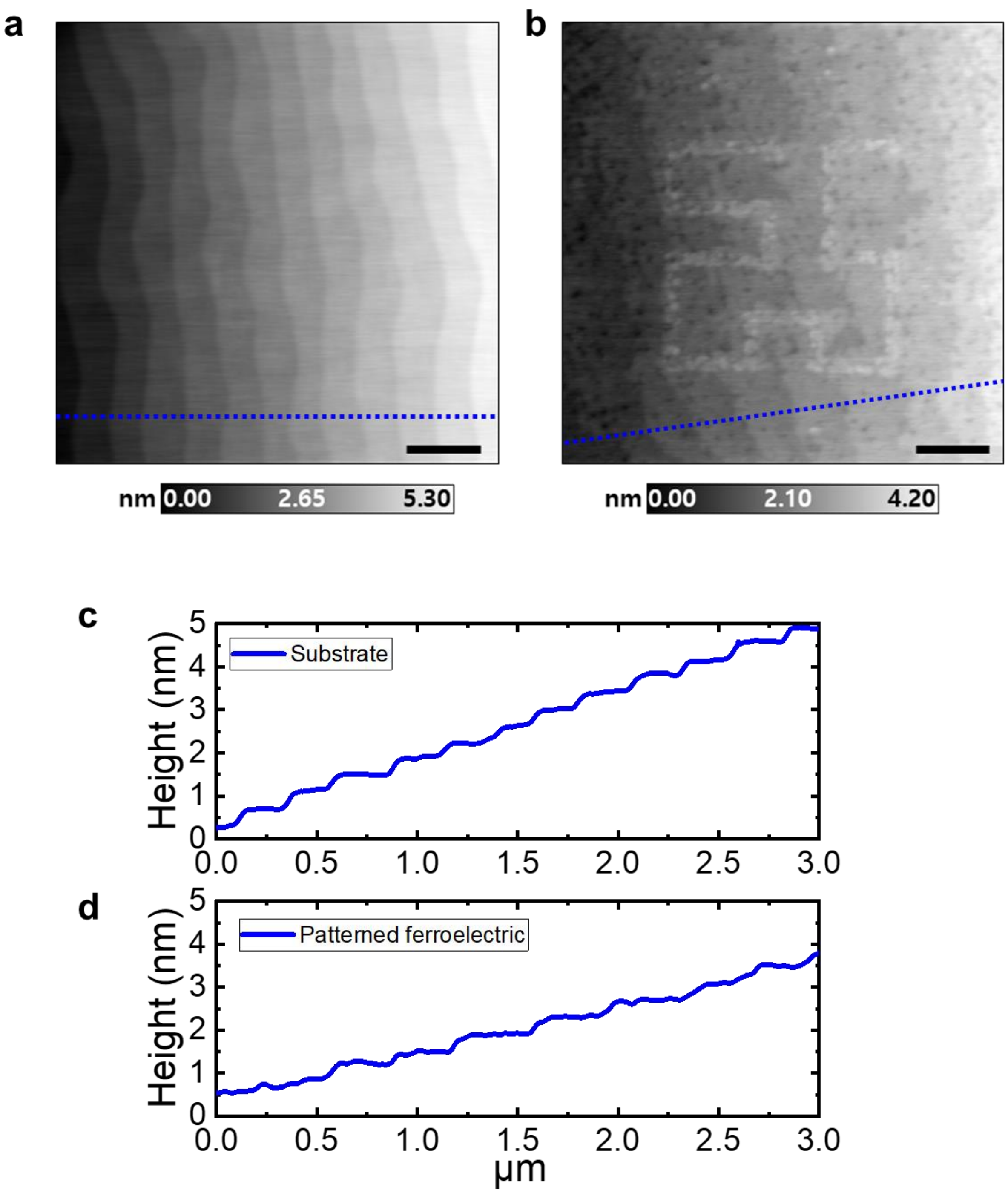

Fig. S32. Lattice-scale polarization-derived lithography. (a) Surface topography of TiO_2-terminated (001)-oriented $SrTiO_3$ substrate and **(b)** patterned $PbTiO_3$ thin film showing atomistic terrace edge features in the bottom of the figures. **(c and d)** Line profiles along the blue lines in (a) and (b). We note that the pristine $PbTiO_3$ surfaces do not exhibit the terrace edge features as shown in Fig. S31, however we observe the features after the appropriate milling scans.

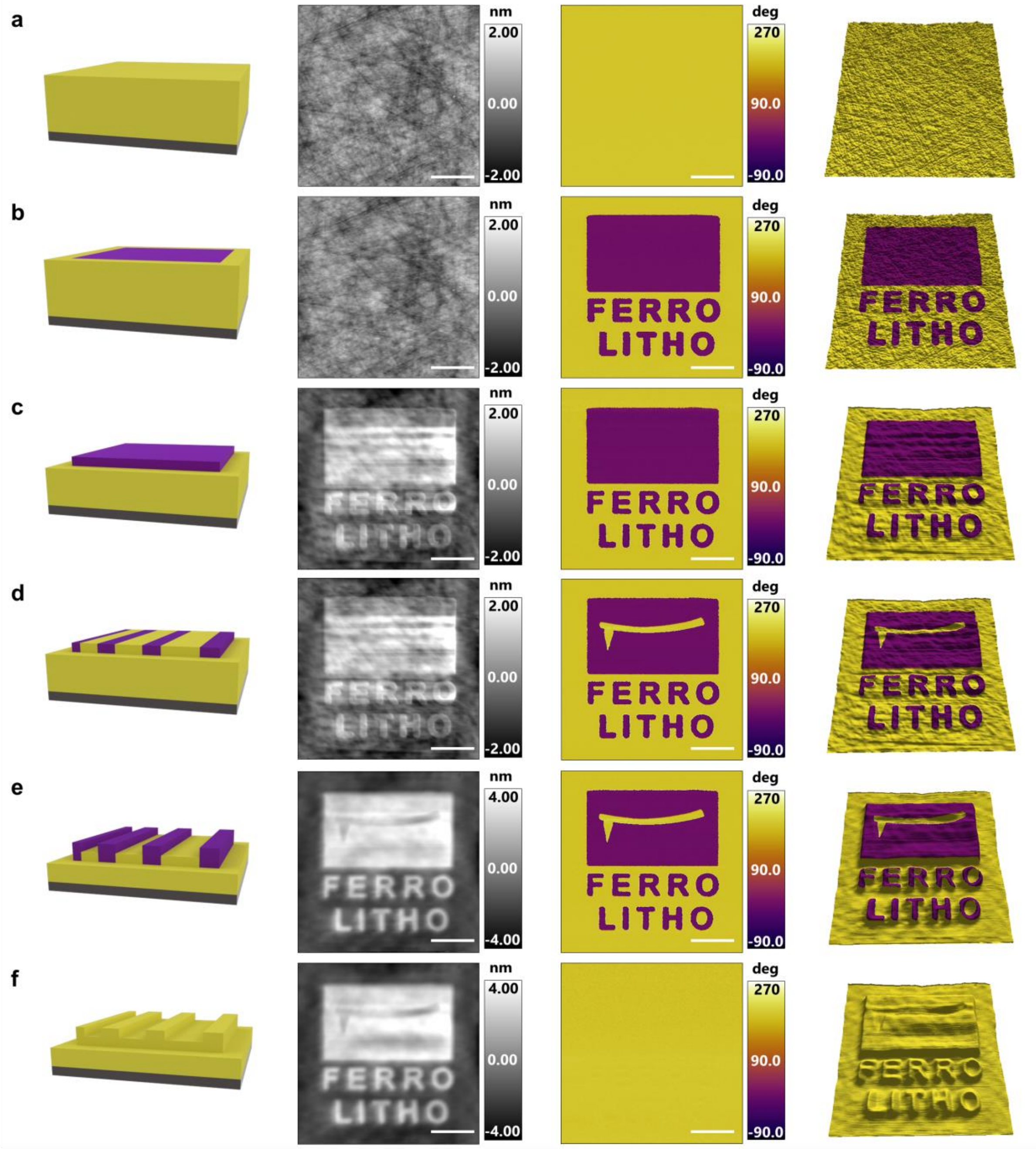

Fig. S33. Demonstration of 3D nanostructure fabrication of a ferroelectric $LiNbO_3$ thin film. Schematic in each step, height, PFM phase and 3D surface superimposed with the PFM phase of **(a)** pristine, **(b)** after first polarization switching, **(c)** after first milling process, **(d)** after second polarization switching, **(e)** after second milling process and **(f)** after switching to uniform up polarization. Scale bars in height and PFM phase images are 3 μm. In 3D images, the scan area is 12.5 μm × 12.5 μm and the z-scale is 20 nm.

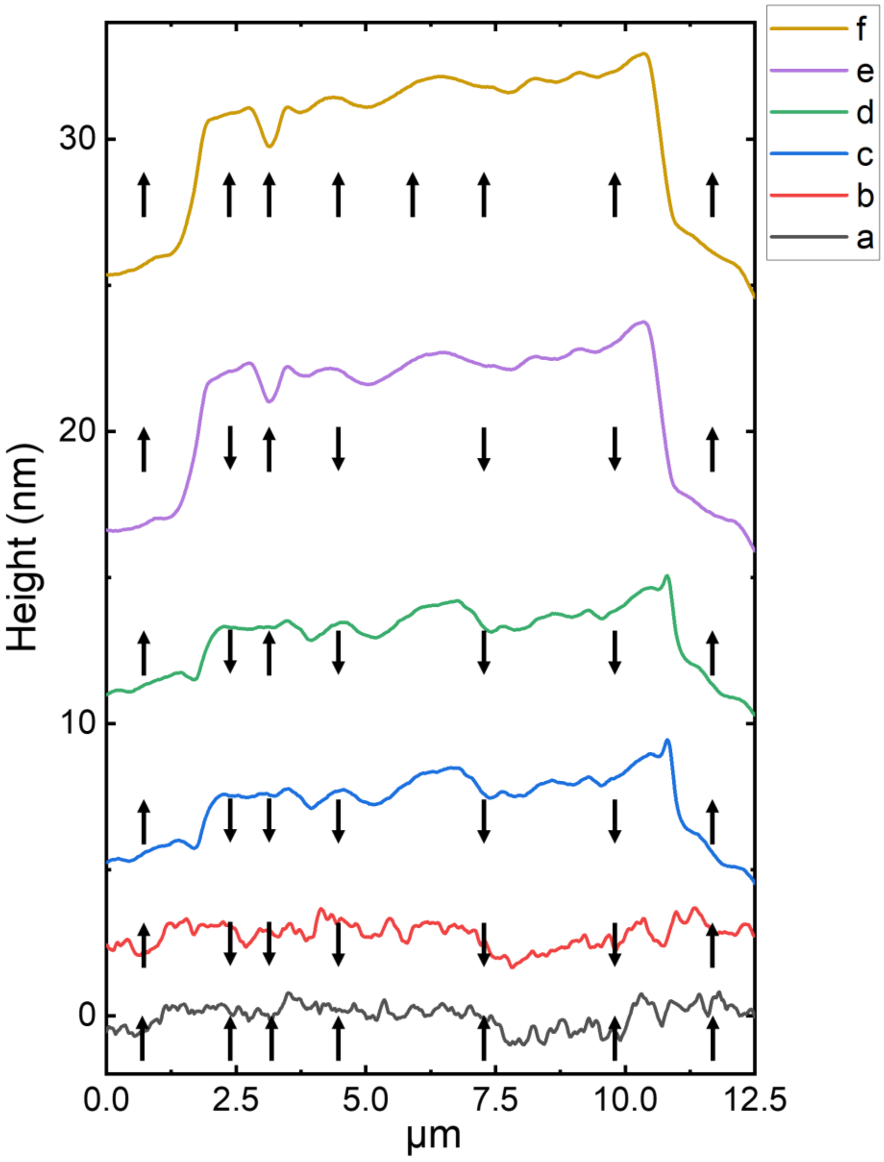

Fig. S34. Structural evolution during the 3D nanostructuring in Fig. S33. The line profiles are obtained in the same position along with the AFM tip feature in Fig. 4b and arrows indicate the domain orientation. Line profiles of **(a)** pristine, (**b)** after 1st polarization switching, **(c)** after 1st milling process, **(d)** after 2nd polarization switching, **(e)** after 2nd milling process and **(f)** after switching to uniform up polarization.

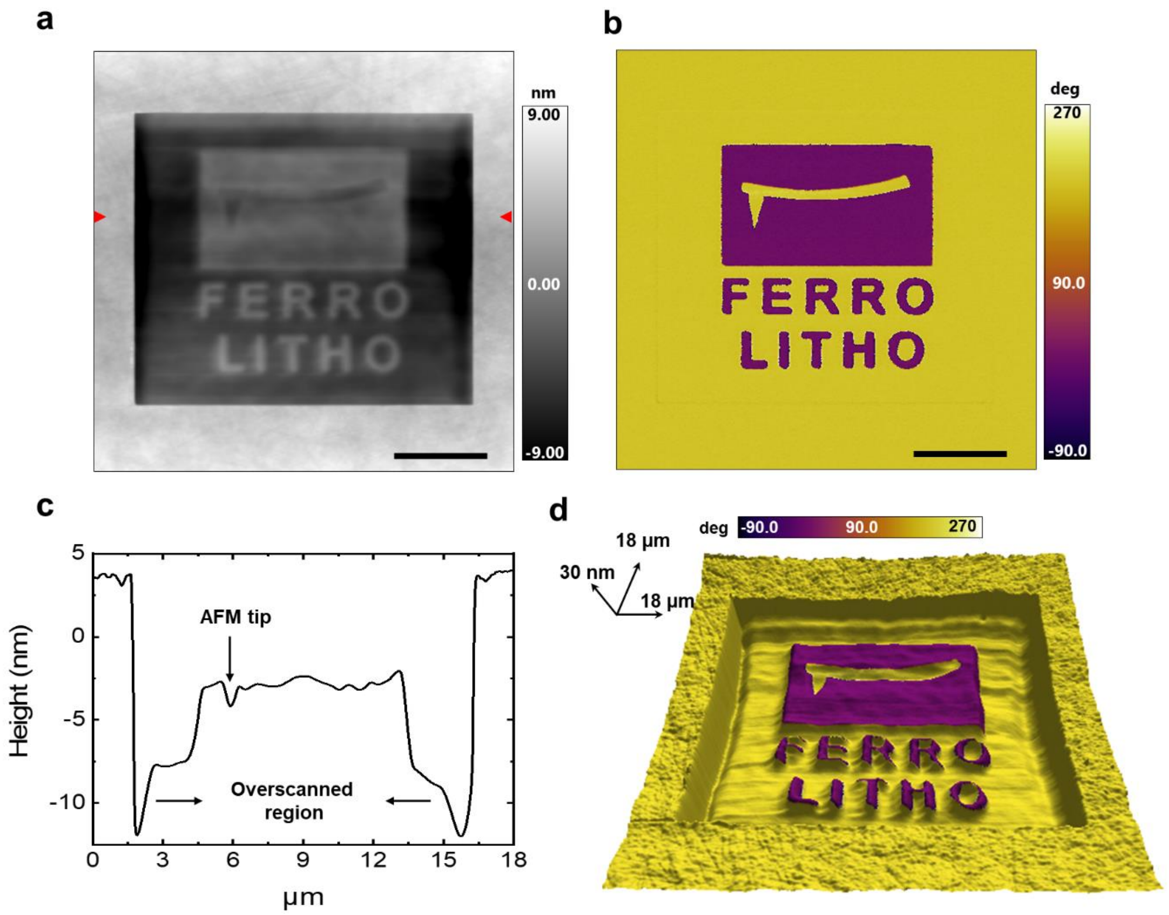

Fig. S35. (a) Height and **(b)** PFM phase with pristine background after 3D nanostructuring. **(c)** Line profile along with the AFM tip feature in (a) (red arrow). **(d)** 3D surface image with color overlapped with its PFM phase. The non-uniform height of the overscanned region originates from the distortion reduction mechanism during the contact imaging of AFM, also described in Fig. S3. The full-length scale along each respective axis is shown with an arrow in (d).

Supplementary Tables

Probe	Description	Spring constant (N/m, nominal)	Asymmetric wear of PPLN
NM-TC	Single crystal diamond (conductive)	350	Observable
DT-NCHR	Diamond-coated (conductive)	80	Observable
CDT-NCHR	Diamond-coated (conductive)	80	Observable
EFM	Pt/Ir-coated (conductive)	2.8	Unobservable (No surface wear)
HQ:NSC16/HARD/ Al BS (DLC-coated)	Diamond-like-carbon-coated (weakly conductive)	40	Observable
D300	Single crystal diamond (non-conductive)	40	Observable
NC-LC	Single crystal diamond (conductive)	125	Observable
Spark 350-Pt	Pt/Ti-coated (conductive)	42	Observable

Table S1. Probe selection for the asymmetric wear of PPLN.

$\boldsymbol{f}$ $[\boldsymbol{V}]$	$\boldsymbol{F_f^{up}/F_f^{down}}$
1	1.0037
10	1.0375
40	1.1095
54	1.1390

Table S2. Ratio of friction in up and down domains for different values of the flexocoupling coefficient. Note that the flexoelectric tensors are isotropic[26]. Flexocoupling coefficients are based on values in Refs. [13–15].

Type of indenter	Load (mN)	Indentation depth (Ferroelectric up, nm)	Indentation depth (Ferroelectric down, nm)
Spherical	2	20.025 ± 1.920	15.807 ± 0.999
Berkovich	2	76.082 ± 1.171	77.645 ± 0.836
Berkovich	5	138.438 ± 1.185	140.629 ± 1.080
Berkovich	10	211.990 ± 2.010	214.488 ± 2.036

Table S3. Indentation depth from loading curves in nanoindentation measurement using two different types of indenters. 15 data points are averaged in each case.

References

1 Stefani, C. *et al.* Mechanical softness of ferroelectric 180° domain walls. *Phys. Rev. X* **10**, 041001 (2020).

2 Codony, D., Mocci, A., Barceló-Mercader, J. & Arias, I. Mathematical and computational modeling of flexoelectricity. *J. Appl. Phys.* **130**, 231102 (2021).

3 Maranganti, R., Sharma, N. & Sharma, P. Electromechanical coupling in nonpiezoelectric materials due to nanoscale nonlocal size effects: Green's function solutions and embedded inclusions. *Phys. Rev. B* **74**, 014110 (2006).

4 Eliseev, E. A., Morozovska, A. N., Glinchuk, M. D. & Blinc, R. Spontaneous flexoelectric/flexomagnetic effect in nanoferroics. *Phys. Rev. B* **79**, 165433 (2009).

5 Mao, S. & Purohit, P. K. Insights into flexoelectric solids from strain-gradient elasticity. *J. Appl. Mech.* **81** (2014).

6 Abdollahi, A. & Arias, I. Phase-field modeling of crack propagation in piezoelectric and ferroelectric materials with different electromechanical crack conditions. *J. Mech. Phys. Solids* **60**, 2100-2126 (2012).

7 Codony, D., Marco, O., Fernández-Méndez, S. & Arias, I. An immersed boundary hierarchical B-spline method for flexoelectricity. *Comput. Methods Appl. Mech. Eng.* **354**, 750-782 (2019).

8 Barceló-Mercader, J., Codony, D., Fernández-Méndez, S. & Arias, I. Weak enforcement of interface continuity and generalized periodicity in high-order electromechanical problems. *Int. J. Num. Methods Eng.* (2021).

9 Fung, Y.-C. A First Course in Continuum Mechanics. *Englewood Cliffs* (1977).

10 Zhao, J. & Pedroso, D. Strain gradient theory in orthogonal curvilinear coordinates. *Int. J. Solids Struct.* **45**, 3507-3520 (2008).

11 Abdollahi, A. & Arias, I. Constructive and destructive interplay between piezoelectricity and flexoelectricity in flexural sensors and actuators. *J. Appl. Mech.* **82**, 121003 (2015).

12 Persson, K. *Materials Data on $LiNbO_3$ (SG:161) by Materials Project.* https://doi.org/10.17188/1184005 (2014).

13 Kogan, S. M. Piezoelectric effect during inhomogeneous deformation and acoustic scattering of carriers in crystals. *Sov. Phys.-Solid State* **5**, 2069-2070 (1964).

14 Zubko, P., Catalan, G. & Tagantsev, A. K. Flexoelectric effect in solids. *Ann. Rev. Mater. Res.* **43** (2013).

15 Cordero-Edwards, K., Kianirad, H., Canalias, C., Sort, J. & Catalan, G. Flexoelectric fracture-ratchet effect in ferroelectrics. *Phys. Rev. Lett.* **122**, 135502 (2019).

16 Piegl, L. & Tiller, W. *The NURBS Book*. (Springer Science & Business Media, 1996).

17 Wriggers, P. & Laursen, T. A. *Computational Contact Mechanics*. Vol. 2 (Springer, 2006).

18 Popov, V. L., Heß, M. & Willert, E. *Handbook of Contact Mechanics: Exact Solutions of Axisymmetric Contact Problems*. (Springer Nature, 2019).

19 Park, J. Y., Ogletree, D., Thiel, P. & Salmeron, M. Electronic control of friction in silicon pn junctions. *Science* **313**, 186-186 (2006).

20 Li, Y., Schmidt, W. & Sanna, S. Defect complexes in congruent $LiNbO_3$ and their optical signatures. *Phys. Rev. B* **91**, 174106 (2015).

21 Cordero-Edwards, K., Domingo, N., Abdollahi, A., Sort, J. & Catalan, G. Ferroelectrics as smart mechanical materials. *Adv. Mater.* **29**, 1702210 (2017).

22 Ashby, M. F. & Cebon, D. Materials selection in mechanical design. *MRS Bull.* **30**, 995 (2005).

23 Esin, A., Akhmatkhanov, A. & Shur, V. Y. Tilt control of the charged domain walls in lithium niobate. *Appl. Phys. Lett.* **114**, 092901 (2019).

24 Kirbus, B. *et al.* Real-time 3D imaging of nanoscale ferroelectric domain wall dynamics in lithium niobate single crystals under electric stimuli: implications for domain-wall-based nanoelectronic devices. *ACS Appl. Nano Mater.* **2**, 5787-5794 (2019).

25 Lu, H. *et al.* Electrical tunability of domain wall conductivity in $LiNbO_3$ thin films. *Adv. Mater*. **31**, 1902890 (2019).

26 Le Quang, H. & He, Q.-C. The number and types of all possible rotational symmetries for flexoelectric tensors. *Proc. R. Soc. A: Math. Phys. Eng. Sci.* **467**, 2369-2386 (2011).
